# DPY30 acts as an ASH2L-specific stabilizer to stimulate the enzyme activity of MLL family methyltransferases on different substrates

**DOI:** 10.1016/j.isci.2022.104948

**Published:** 2022-08-16

**Authors:** Lijie Zhao, Naizhe Huang, Jun Mencius, Yanjing Li, Ying Xu, Yongxin Zheng, Wei He, Na Li, Jun Zheng, Min Zhuang, Shu Quan, Yong Chen

**Affiliations:** 1 State Key Laboratory of Molecular Biology, National Center for Protein Science Shanghai, Shanghai Institute of Biochemistry and Cell Biology, Center for Excellence in Molecular Cell Science, Chinese Academy of Sciences, Shanghai 200031, China; 2 University of Chinese Academy of Sciences, Beijing 100049, China; 3 State Key Laboratory of Bioreactor Engineering, East China University of Science and Technology, Shanghai Collaborative Innovation Center for Biomanufacturing (SCICB), Shanghai 200237, China; 4 National Facility for Protein Science in Shanghai, Zhangjiang Lab, Shanghai Advanced Research Institute, Chinese Academy of Science, Shanghai 201210, China; 5 School of Life Science and Technology, ShanghaiTech University, 100 Haike Road, Shanghai 201210, China; 6 Shanghai Frontiers Science Center of Optogenetic Techniques for Cell Metabolism, Shanghai 200237, China

**Keywords:** Biological sciences, Biochemistry, Biocatalysis, Bioengineering

## Abstract

Dumpy-30 (DPY30) is a conserved component of the mixed lineage leukemia (MLL) family complex and is essential for robust methyltransferase activity of MLL complexes. However, the biochemical role of DPY30 in stimulating methyltransferase activity of MLL complexes remains elusive. Here, we demonstrate that DPY30 plays a crucial role in regulating MLL1 activity through two complementary mechanisms: A nucleosome-independent mechanism and a nucleosome-specific mechanism. DPY30 functions as an ASH2L-specific stabilizer to increase the stability of ASH2L and enhance ASH2L-mediated interactions. As a result, DPY30 promotes the compaction and stabilization of the MLL1 complex, consequently increasing the HKMT activity of the MLL1 complex on diverse substrates. DPY30-stabilized ASH2L further acquires additional interfaces with H3 and nucleosomal DNA, thereby boosting the methyltransferase activity of the MLL1 complex on nucleosomes. These results collectively highlight the crucial and conserved roles of DPY30 in the complex assembly and activity regulation of MLL family complexes.

## Highlights

• DPY30 stimulates the enzyme activity of MLL complexes on broad-spectrum substrates
• DPY30 functions as an ASH2L-specific stabilizer
• DPY30 promotes the compaction and stabilization of the MLL1 complex
• DPY30-stabilized ASH2L acquires additional interfaces with H3 and nucleosomal DNA

## Graphical abstract

**Figure:**
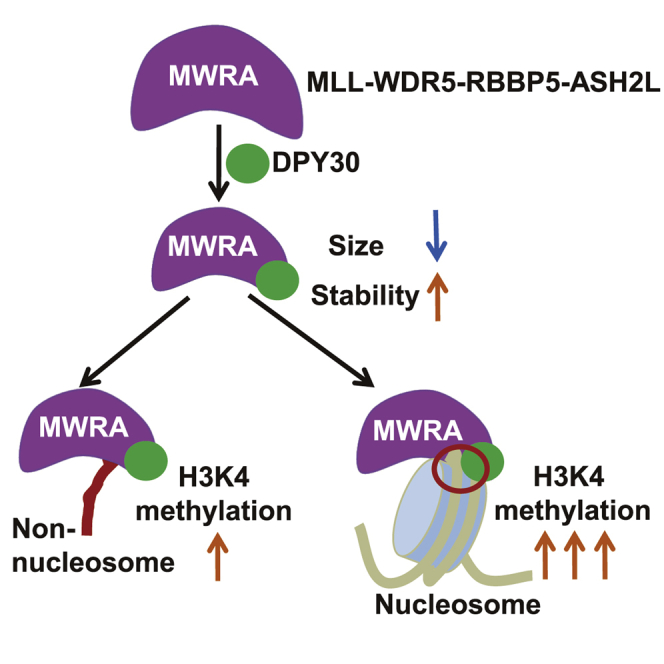

## Introduction

H3K4 methylation is critical to the epigenetic regulation of gene transcription (Hyun et al., 2017; Shilatifard, 2008). Defects in H3K4 methylation have been closely associated with a broad spectrum of hematologic and solid malignancies (Rao and Dou, 2015; Yang and Ernst, 2017). H3K4 methylation is mainly mediated by MLL family proteins, including MLL1, MLL2, MLL3, MLL4, SET1A, and SET1B (Ansari and Mandal, 2010). Among them, MLL1 has drawn the most attention because its chromosomal translocations lead to various forms of acute lymphoid and myeloid leukemia (Krivtsov et al., 2017).

Histone lysine methylation by MLL family proteins is catalyzed by their C-terminal SET (Su(var)3–9, Enhancer of zeste, and Trithorax) domains, but the intrinsic catalytic activity of SET domains in MLL family proteins (MLL_SET_) is relatively low (Dou et al., 2006). The robust enzymatic activity of MLL_SET_ requires the formation of a multimeric complex composed of MLL_SET_, WD Repeat containing protein 5 (WDR5), Retinoblastoma-Binding Protein 5 (RBBP5), Absent, Small, or Homeotic 2-Like (ASH2L), and Dumpy-30 (DPY30), collectively referred to as ‘MWRAD’ (Couture and Skiniotis, 2013; Patel et al., 2009). The molecular mechanism of activity regulation by WDR5, RBBP5, and ASH2L has been elucidated by structural studies of MLL complexes (Han et al., 2019; Li et al., 2016; Park et al., 2019; Steward et al., 2006; Xue et al., 2019). However, the exact role of DPY30 in the regulation of MLL activity remains unclear.

Dpy-30 was initially discovered in *Caenorhabditis elegans* as a regulator in X chromosome dosage compensation (Hsu and Meyer, 1994). Mammalian DPY30 can be assembled into MLL family complexes through its C-terminal 44-residue helical bundle, termed the docking and dimerization domain (DD domain), which directly interacts with the ASH2L C-terminal DPY30-binding motif (DBM) (Haddad et al., 2018; South et al., 2010). In embryonic stem cells (ESCs), knockdown of DPY30 reduces H3K4 trimethylation and impairs ESC plasticity in transcriptional reprogramming *in vivo* (Jiang et al., 2011). Similarly, knockdown of DPY30 by siRNA led to decreased H3K4 methylation levels and inhibited the proliferation and differentiation of hematopoietic progenitor cells (Yang et al., 2014). Complete depletion of DPY30 from conditional knockout mice resulted in severely decreased H3K4me1/me2/me3 in bone marrow cells (Yang et al., 2016), demonstrating that DPY30 is essential for the optimal HKMT activity of MLL family complexes *in vivo*.

How DPY30 biochemically contributes to the H3K4 methylation process remains controversial. Early studies showed that DPY30 was dispensable for the HKMT activity of the MLL1 complex when using recombinant histone H3 or H3 peptides as substrates (Haddad et al., 2018; Kwon et al., 2020). Recent studies revealed that DPY30 dramatically stimulated the HKMT activity of MLL complexes on chromatin and nucleosome core particles (NCPs) but not on free H3 (Kwon et al., 2020; Lee et al., 2021). The role of DPY30 was proposed to restrain the MLL rotation dynamics of MLL on NCP (Lee et al., 2021). However, the essential role of DPY30 in ensuring HKMT activity on NCP is not manifested by the solved cryo-EM structures of MWRAD-NCP complexes. In the MLL1-WDR5-RBBP5-ASH2L-DPY30-NCP(M1WRAD-NCP) and MLL3-WDR5-RBBP5-ASH2L-DPY30-NCP (M3WRAD-NCP) structures reported by Xue et al., the electron density of DPY30 cannot be observed (Xue et al., 2019). In another cryo-EM structure of M1WRAD-NCP reported by Park et al. (2019), DPY30 was present in a very small fraction (3.3%) of the complex and only reconstituted at the lowest resolution in the whole complex (10-12 Å), which hardly solved the secondary structures of ASH2L or DPY30 and could not reveal the interaction details between DPY30 and other proteins. These structural studies suggest that DPY30 is flexibly associated with the rest of the MLL complex. These apparent contradictions prompted us to investigate how DPY30 is integrated into the MLL1 complex and contributes to the methylation activity of the MLL1 complex on nucleosomes or other non-nucleosome substrates.

In this work, we reveal that DPY30 can stimulate the HKMT activity of the MLL1 complex through a nucleosome-independent mechanism and a nucleosome-specific mechanism. By combining crosslinking mass spectrometry (CX-MS), structural prediction, molecular dynamics (MD) analyses, biological small-angle X-ray scattering (SAXS), and biochemical assays, we demonstrate that DPY30 functions as an ASH2L-specific stabilizer. DPY30-stabilized ASH2L acquires functional improvement to enhance ASH2L-mediated interactions to promote the assembly and stabilization of the MLL1 complex, which explains the activity stimulation on a wide range of substrates (H3 peptide, H3/H4 tetramer, and octamer) by DPY30. DPY30-stabilized ASH2L further gains additional interfaces with nucleosomal DNA and H3, thereby specifically boosting HKMT activity on nucleosomes.

## Results

### DPY30 enhances the HKMT activity of the MLL1 complex

**Figure 1:**
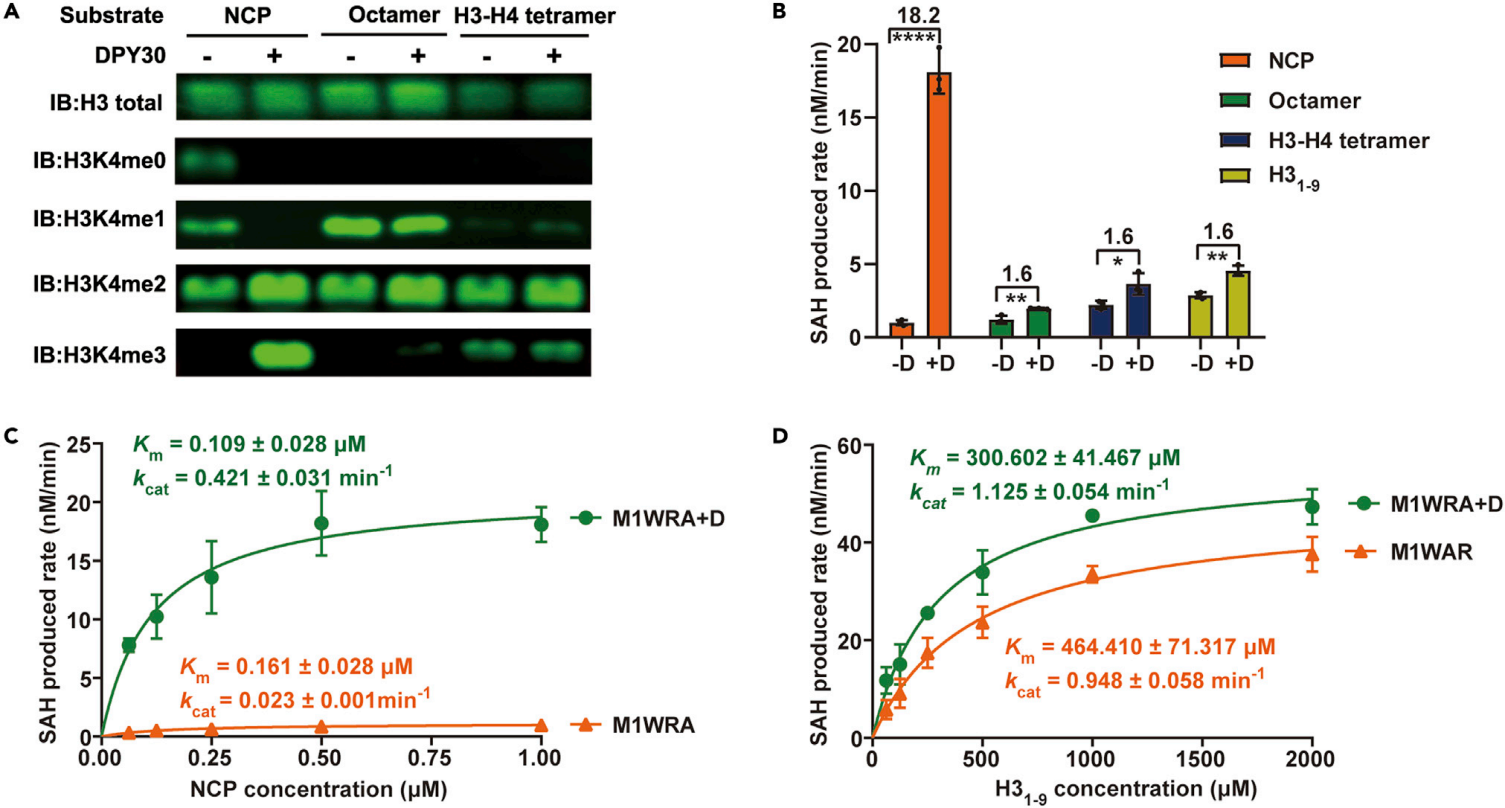
DPY30 enhances the HKMT activity of the MLL1 complex (A) Comparison of the methylation activities of M1WRA and M1WRA + D on NCP, histone octamer, and H3-H4 tetramer. The reaction system containing MLL1 complex (2 μM), substrate (2 μM), and SAM (100 μM) was incubated at 30°C for 30 min. Samples were separated by 12% SDS–PAGE and blotted with anti-H3C (representing the total H3 amount), H3K4me0, H3K4me1, H3K4me2, and H3K4me3 antibodies as indicated on the left. (B) Methylation rates of M1WRA and M1WRA + D on NCP, Octamer, H3-H4 tetramer, and H3_1-9_ measured by an MTase-Glo Methyltransferase Assay kit. The reaction system contained MLL1 complexes (50 nM), substrate (40 μM for H3_1-9_, 1 μM for the other three substrates), and SAM (40 μM). “+D” indicates the mixing of DPY30 and the preassembled MWRA complex. Fold changes and statistical significance are indicated. Data are shown as the mean ± SD from three independent measurements. (C) Michaelis–Menten kinetic analyses of HKMT activities of M1WRA and M1WRA + D complexes on NCP. The *k*_cat_ and *K*_m_ are shown as the mean ± SD from three independent experiments. (D) Michaelis–Menten kinetic analyses of HKMT activities of M1WRA and the M1WRA + D complex on H3_1-9_.

To examine the role of DPY30 in stimulating MLL1 activity, we first characterized the HKMT activity of the MLL1 complex (MLL1-WDR5-RBBP5-ASH2L, abbreviated as M1WRA) in the presence and absence of DPY30 on different substrates by performing a western-blot-based methyltransferase assay. We found that DPY30 remarkably enhanced the HKMT activity of the MLL1 complex on NCPs but had negligible effects on octamer and H3-H4 tetramer substrates (Figure 1A). To quantitatively dissect the roles of DPY30 on different substrates, we compared the reaction rates of MLL1 core complexes by using an MTase-Glo Methyltransferase Assay kit. DPY30 substantially boosted the methylation rate on NCP by ∼18-fold but only increased the reaction rate on the octamer, H3-H4 tetramer, and H3_1-9_ peptide by ∼1.6-fold Figure 1B).

To further compare the kinetic difference of the MLL1 complex in the presence or absence of DPY30 on different substrates, we performed steady-state kinetic analyses of the MLL1 complex by fixing the concentration of AdoMet and changing the substrate concentrations of NCP or H3_1-9_. In the absence of DPY30, M1WRA exhibited relatively weak activity with a *K*_m_ (NCP) of 0.16 μM and a turnover rate (*k*_cat_) of 0.023 min^−1^. In the presence of DPY30, the MLL1 complex (M1WRA + D) exhibited strikingly boosted activity on NCP with an ∼18-fold increase in *k*_cat_and a 1.5-fold decrease in *K*_m_ (Figure 1C). Thus, for NCP substrates, the catalytic efficiency (*k*_cat_/*K*_m_) of the MLL1 complex with DPY30 was 27-fold higher than that of the MLL1 complex without DPY30. In contrast, the activity-stimulating effect of DPY30 was much weaker on the H3_1-9_ peptide, with a 1.2-fold increase in *k*_cat_ and a 1.5-fold decrease in *K*_m_ (Figure 1D). The 1.8-fold increase in the catalytic efficiency (*k*_cat_/*K*_m_) of the MLL1 complex indicates a weak but appreciable effect of DPY30 in stimulating the activity of the MLL1 complex on H3 peptides. These data not only reveal the important role of DPY30 in ensuring optimal HKMT activity of the MLL1 complex but also suggest that DPY30 could stimulate HKMT activity through a nucleosome-independent mechanism and a nucleosome-specific mechanism.

### The activity-stimulating effect of DPY30 is dependent on ASH2L

We sought to explore the underlying mechanism by which DPY30 stimulates HKMT activity of the MLL1 complex. Previous studies have reported that DPY30 only interacts with ASH2L in the MLL1 complex by binding to the C-terminal DBM of ASH2L (Haddad et al., 2018; Patel et al., 2009). We first investigated whether the activity-stimulating role of DPY30 is dependent on its interaction with ASH2L. By utilizing an ASH2L mutant (L513E/L517E/V520E) (hereafter referred to as ASH2L^3E^) that completely abolished the interaction with DPY30 (Chen et al., 2012), as shown in the GST pull-down assay (Figure S1A), we found that DPY30 could not increase the HKMT activity of the MLL1 complex reconstituted with this DPY30-binding-deficient ASH2L mutant on NCPs or H3 peptides (Figures S1B and S1C). Moreover, the DPY30 dimerization domain (DPY30_DD_, 45-99), mediating the interaction with ASH2L (Haddad et al., 2018), was sufficient and necessary to stimulate the HKMT activity of the MLL1 complex (Figure S1B). A previously-identified DPY30 mutant (L69D), which impairs DPY30 dimerization and abolishes its binding to ASH2L (Figure S1D) (Tremblay et al., 2014), could not increase the HKMT activity of the MLL1 complex (Figures S1B). These results reveal that the stimulatory effect of DPY30 relies on its interaction with ASH2L.

**Figure 2:**
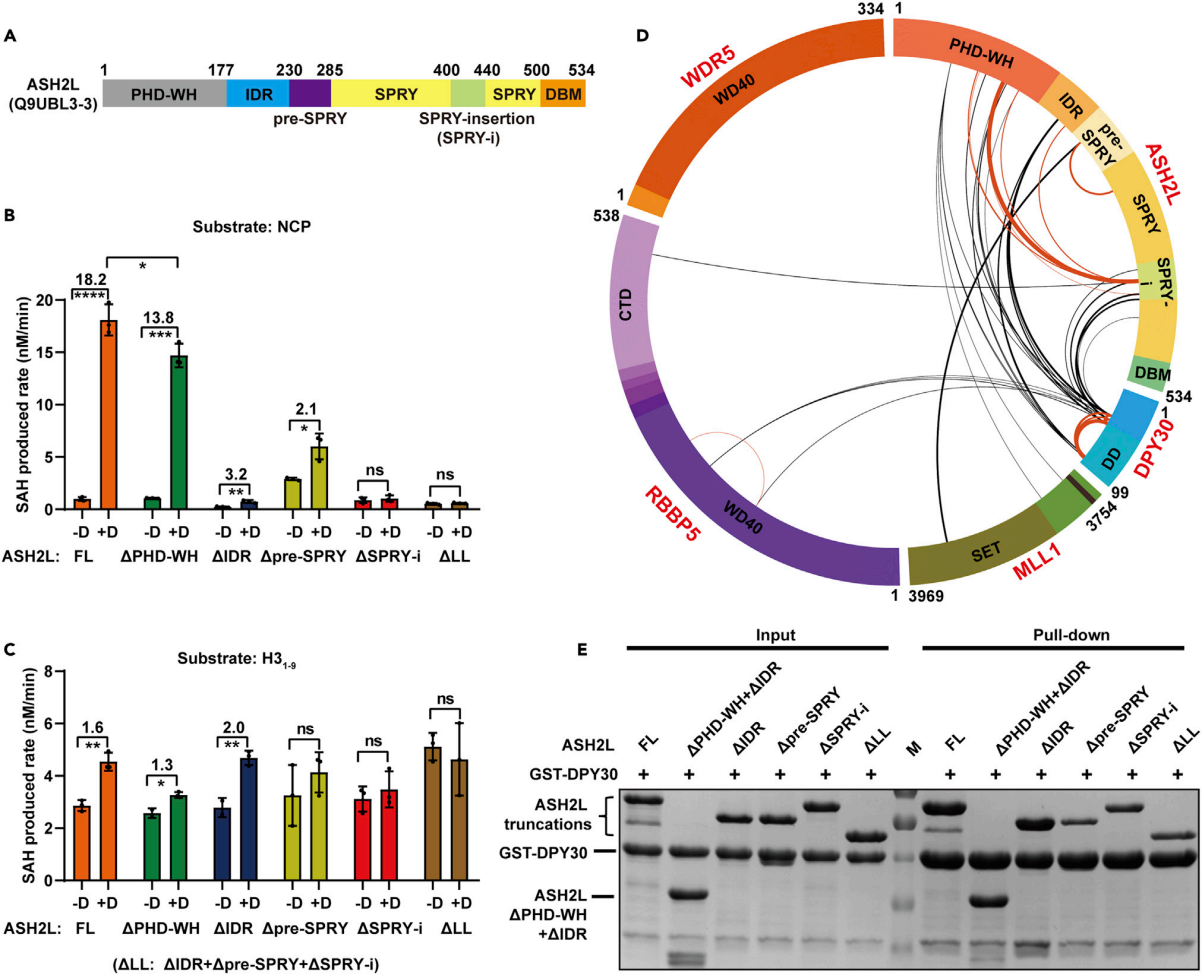
DPY30 interacts with multiple regions of ASH2L (A) Domain organization of ASH2L (UniProt ID: Q9UBL3-3). PHD-WH, plant homeodomain-winged helix; IDR, intrinsically disordered region; SPRY, splA and ryanodine receptor domain; DBM, DPY30 binding motif. (B and C) Comparison of the DPY30 effect on methylation rates of MLL1 complexes assembled with different ASH2L truncations on NCP (B) and H3_1-9_ (C) measured by the MTase-Glo Methyltransferase Assay kit. FL: full length. ΔLL: deletion of IDR, pre-SPRY, and SPRY-insertion motifs. Fold changes and statistical significance are indicated. Data are shown as the mean ± SD from three independent measurements. (D) Circular plot of specific crosslinks detected in M1WRAD but not in M1WRA. Intrasubunit crosslinks are shown in orange, and intersubunit crosslinks are shown in black. The thickness of the line is correlated with the spectrum number of the crosslinked peptide detected. (E) GST pull-down assays revealed that multiple regions of ASH2L were involved in DPY30 binding. Different ASH2L truncations are indicated on the top. See also Figures S1–S3 and Table S1.

Next, we investigated which ASH2L domain(s) contribute to DPY30-induced stimulation of MLL1 activity. ASH2L encompasses an N-terminal PHD-WH domain (1-177), an intrinsically disordered region (IDR) (178-229), a pre-SPRY motif (230-285), a split SPRY domain (286-499) with an SPRY-insertion (400-440), and a C-terminal DPY30-binding motif (DBM, 500-534) (Figure 2A). The essential roles of the ASH2L SPRY domain in the regulation of MLL1 complex activity have been firmly established (Li et al., 2016), but the functions of other ASH2L domains remain elusive. We used a series of ASH2L constructs with the deletion of each domain or motif to probe how these individual domains or motifs affect the DPY30-dependent activity stimulation of the MLL1 complex. All ASH2L domain-deletion mutants were eluted at the expected peak positions corresponding to their molecular weights on gelfiltration chromatography and could be assembled into M1WRA complexes (Figures S2A–S2C), suggesting that these domain-deletion mutants retained the structural integrity of ASH2L. To our surprise, all ASH2L domains were required for maintaining the optimal HKMT activity of the MLL1 complex. Deleting the PHD-WH domain slightly reduced the stimulatory effect of DPY30, whereas deletion of IDR, pre-SPRY, and SPRY-insertion severely decreased the stimulatory effects of DPY30 on NCP substrates (Figure 2B). Combined deletion of IDR, pre-SPRY, and SPRY-insertion motifs (ΔLL) completely abolished DPY30-dependent activity stimulation on NCP (Figure 2B). Notably, deletion of pre-SPRY or SPRY-insertion, but not PHD-WH or IDR, also disrupted the stimulatory effect of DPY30 on H3_1-9_ peptides (Figure 2C). These results indicate that these previously noteless domains or motifs of ASH2L play essential roles to enhance the HKMT activity of the MLL1 complex: the pre-SPRY and SPRY-insertion are critical for both nucleosome-independent and nucleosome-specific activity stimulation, whereas PHD-WH and IDR are only required for nucleosome-specific activity stimulation by DPY30.

### DPY30 interacts with multiple regions of ASH2L

The functional interplay between DPY30 and these less-characterized motifs of ASH2L intrigued us to re-examine the DPY30-mediated interactions in the MLL1 complex. We performed crosslinking mass spectrometry (CX-MS) to probe the potential differences in protein–protein interaction networks of the MLL1 complex induced by DPY30. By using a cutoff of spectrum counts of more than three and an E score value smaller than 0.02, we identified 158 crosslinked peptides in the MLL1 complex with DPY30 (M1WRAD) and 122 crosslinked peptides in the MLL1 complex without DPY30 (M1WRA) (Data S1). The majority of the crosslinked peptides were shared in the two complexes, but the addition of DPY30 induced 47 specific crosslinks identified exclusively in the M1WRAD complex (Figure S3A and Table S1). DPY30 is intensely crosslinked to the SPRY-insertion, IDR, and PHD-WH domains of ASH2L (Figure 2D). It should be noted that no crosslink was detected between DPY30 and ASH2L_DBM_ because there is no lysine residue in the ASH2L_DBM_ region to enable crosslinking. In addition to the newly found DPY30-ASH2L intermolecular crosslink, we also found that DPY30 induced a substantial enrichment of the intramolecular crosslinks in ASH2L, especially the extensive crosslinks between SPRY-insertion and other regions of ASH2L (Figure 2D).

The widespread crosslinks between multiple regions of ASH2L and DPY30, as well as between RBBP5 and DPY30, indicate that these ASH2L or RBBP5 regions may interact with DPY30 or be in closeness with DPY30. To distinguish these two possibilities, we performed GST pull-down assays to characterize DPY30 interactions with ASH2L or RBBP5. No interaction was detected between DPY30 and RBBP5 under our assay conditions (Figure S3B), indicating that DPY30 and RBBP5 may just be in spatial proximity but do not directly interact with each other. In sharp contrast, ASH2L was readily pulled down by GST-DPY30 (Figure 2E). Moreover, we found that deletion of PHD-WH and IDR did not affect the amount of ASH2L pulled down by GST-DPY30, but the deletion of pre-SPRY or SPRY-insertion severely decreased the ASH2L-DPY30 interactions (Figures 2E and S3C), suggesting that pre-SPRY and SPRY-insertion of ASH2L may be directly involved in the interaction with DPY30.

### The structural basis of the interaction between DPY30 and ASH2L

**Figure 3:**
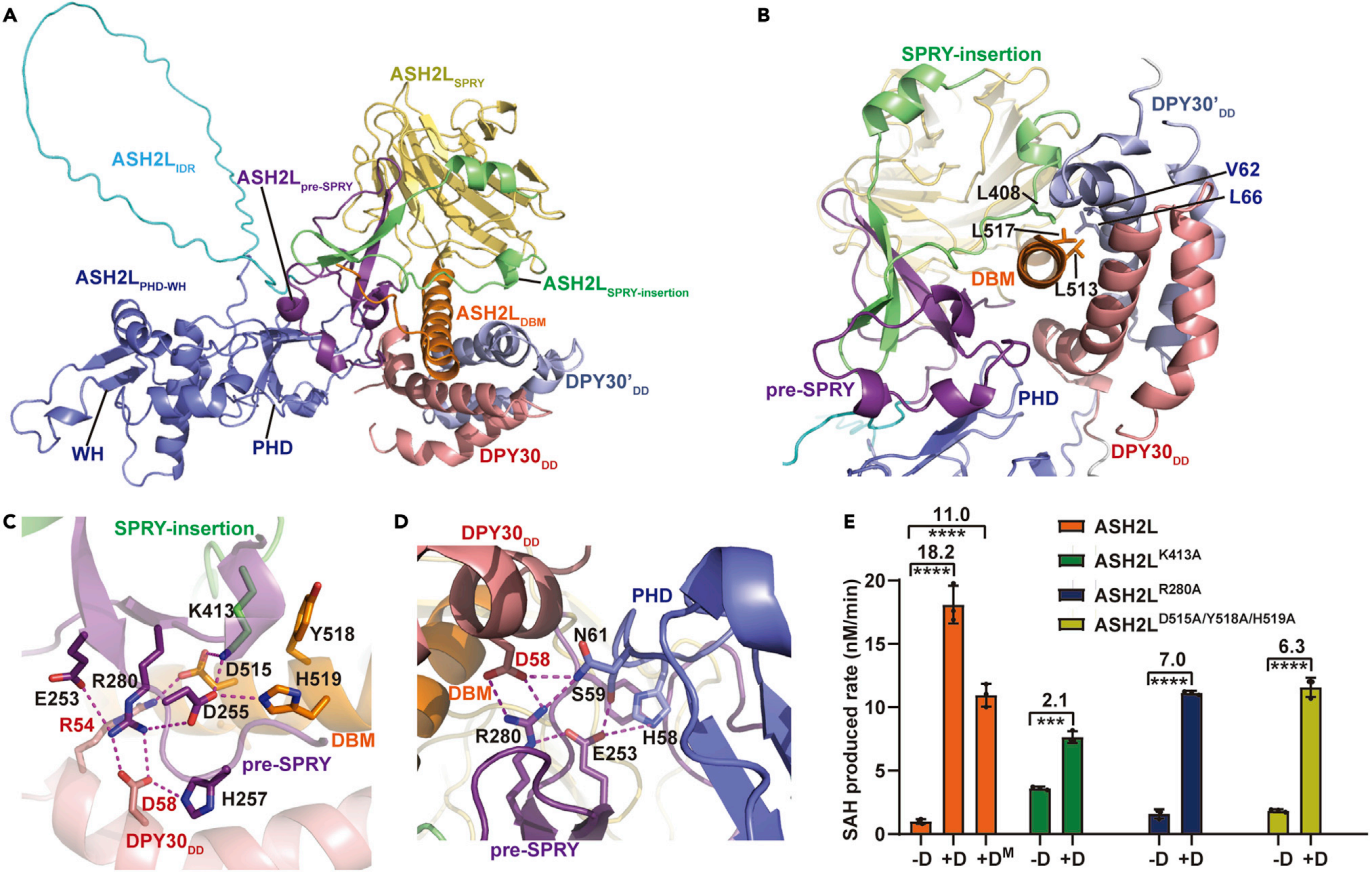
The structural basis of the interaction between DPY30 and ASH2L (A) The predicted structure of the ASH2L-DPY30 complex. Different ASH2L domains or motifs are in the same color codes as in Figure 2A: ASH2L_PHD-WH_, dark blue; ASH2L_IDR_, cyan; ASH2L_pre-SPRY_, magenta; ASH2L_SPRY_, yellow; ASH2L_SPRY-insertion_, green; ASH2L_DBM_, orange. Two DPY30 are colored in light blue and red, respectively. (B) The hydrophobic interface between ASH2L and DPY30. ASH2L_pre-SPRY_, ASH2L_SPRY-insertion_, and two DPY30 embrace ASH2L_DBM_. ASH2L_PHD_ also contacts ASH2L_pre-SPRY_ and one copy of DPY30. The critical hydrophobic residues are presented as stick models. (C) Electrostatic interaction interface between ASH2L and DPY30. The critical residues are shown as stick models. Hydrogen bonds are shown as dashed magenta lines. (D) The interface among ASH2L_PHD_, ASH2L_pre-SPRY_, and DPY30. The critical residues are shown as stick models. Hydrogen bonds are shown as dashed magenta lines. (E) ASH2L and DPY30 mutations at the interaction interface decreased the activity-stimulating effect of DPY30 on NCP. D^M^ is a DPY30 mutant (R54A/D58A). See also Figures S4–S6.

Next, we sought to determine how pre-SPRY and SPRY-insertion of ASH2L contribute to DPY30 binding. After extensive but unsuccessful attempts to crystallize the ASH2L-DPY30 complex, we decided to use AlphaFold2 to predict the ASH2L-DPY30 complex structure and apo ASH2L structure (Figures 3A and S4A) (Mirdita et al., 2022). ASH2L exhibits a two-lobe structure separated by a flexible IDR: one lobe (aa 1-177) is the PHD-WH domain, and the other lobe (aa 230-534) is composed of pre-SPRY, SPRY, SPRY-insertion, and DBM motifs (Figure S4A). Each lobe shows a compact fold with high pLDDT (prediction local distance difference test) values, indicating high confidence of structural prediction, but the ASH2L_IDR_ loop (178-229) connecting the two lobes does not have any defined structure (Figure S4B). As a result, the PHD-WH domains show random orientations relative to ASH2L_230-534_ in five apo ASH2L models (Figure S4C). The ASH2L-DPY30 complex also shows a two-lobe feature (Figures S4D and S4E). Although ASH2L_IDR_ is still unstructured, the PHD-WH domain in the ASH2L-DPY30 complex has a fixed orientation relative to ASH2L_230-534_ (Figure S4F). The PAE (predicted aligned error) plots also confirmed that the ASH2L_PHD-WH_ domain has some inter-domain packings with ASH2L pre-SPRY, SPRY-insertion, DBM, and one DPY30 (Figure S4G). This suggests that DPY30 binding restrains the rotational freedom between ASH2L_1-177_ and ASH2L_230-534_. This notion was supported by the observation that DPY30 induced more intramolecular crosslinked peptides of ASH2L in the M1WRAD complex, especially dramatically increased crosslinking peptides between PHD-WH and SPRY-insertion (Figure 2D).

A recent publication reported that the pre-SPRY and SPRY-insertion motifs of ASH2L are intrinsically disordered regions (IDRs), and DPY30 induces conformational changes in ASH2L IDRs to form ordered structures (Lee et al., 2021). However, the structural prediction of apo ASH2L indicates that pre-SPRY and SPRY-insertion motifs have well-defined structural features with high confidence (Figures S4A and S4B). The structures of the apo ASH2L_230-534_ and ASH2L_230-534_-DPY30 complexes can be superimposed with a root-main-square deviation (rmsd) value of 0.32 Å (Figure S4H). The pre-SPRY and SPRY-insertion motifs in both structures are almost identical. Molecular dynamic simulation analyses further indicated that the secondary structures of pre-SPRY and SPRY-insertion motifs in both apo ASH2L and ASH2L-DPY30 remained stable during the simulation process (Figures S5A–S5D), suggesting that DPY30 may not directly induce the conformational change of ASH2L_pre-SPRY_ and ASH2L_SPRY-insertion,_ as proposed by the previous publication (Lee et al., 2021)

In the representative complex structure model, the main DPY30-ASH2L binding interface is established by the ASH2L DBM helix docked into the hydrophobic cleft of DPY30 dimers (Figure 3B). The pre-SPRY and the SPRY-insertion motifs of ASH2L function as two arms to clamp the ASH2L DBM helix, thus embracing the DBM helix together with two DPY30 dimerization helices (Figure 3B). Consistent with the structural model, deletion of pre-SPRY and SPRY-insertion decreased the interaction between ASH2L and DPY30 (Figure 2E). In addition, the ASH2L PHD domain directly contacts ASH2L_pre-SPRY_ and one copy of DPY30 (Figure 3B).

The importance of pre-SPRY and SPRY-insertion in maintaining the ASH2L-DPY30 interaction can also be inferred from the structural comparison of our AlphaFold2 model with previously determined ASH2L-DPY30 structures, including the ASH2L_SPRY_-DPY30 crystal structure (PDB: 6E2H) (Haddad et al., 2018), ASH2L-DPY30 from the M1WRAD-NCP cryo-EM structure (PDB: 6PWV) (Park et al., 2019), and yeast Bre2-Sdc1 (ASH2L-DPY30 homolog) structures (PBD: 6CHG, 6VEN) (Hsu et al., 2018; Worden et al., 2020) (Figures S6A). All these structures showed the conserved ASH2L_DBM_-DPY30 interface (Figure S6A). Notably, because of the lack of the pre-SPRY and SPRY-insertion motifs in the designed ASH2L_SPRY_ construct, the SPRY domain in the ASH2L_SPRY_-DPY30 crystal structure (PDB: 6E2H) rotated approximately 90° relative to the SPRY domains in other complex structures superimposed by DBM helices (Figure S6A), indicating that the inclusion of pre-SPRY and SPRY-insertion motifs ensures the conserved ASH2L-DPY30/Bre2-Sdc1 binding mode in different species. In addition, the predicted ASH2L-DPY30 structure can be superimposed into the M1WRAD-NCP structure without any clash with other MLL complex or NCP components (Figure S6B), suggesting that the similar ASH2L-DPY30 configuration could be maintained in the M1WRAD-NCP complex.

A complex array of electrostatic interactions stabilizes the tetrapartite interface composed of ASH2L_pre-SPRY_, ASH2L_SPRY-insertion_, ASH2L_DBM_, and DPY30_DD_ (Figure 3C). For example, DPY30^D58^ forms three saltbridges with H257 and R280 from ASH2L_pre-SPRY_, and R280 is further secured by electrostatic interactions with D255, which additionally coordinates two positively charged residues, including K413 from ASH2L_SPRY-insertion_ and H519 from ASH2L_DBM_. DPY30^R54^ is linked to K413 from ASH2L_SPRY-insertion_ through the D515 bridge from ASH2L_DBM_ (Figure 3C). In addition, the PHD domain in ASH2L_PHD-WH_ interacts with pre-SPRY and DPY30 mainly through electrostatic and hydrogen-bonding interactions (Figure 3D). In support of the important roles of these electrostatic interactions, mutations of ASH2L R280A, K413A, D515A/Y518A/H519A, or DPY30 R54A/D58A, which did not affect the structural integrity of ASH2L (Figure S6C), specifically decreased the activity stimulation by DPY30 (Figure 3E). Collectively, these results reveal some previously unrecognized interaction interfaces between ASH2L and DPY30.

### DPY30 stabilizes ASH2L

**Figure 4:**
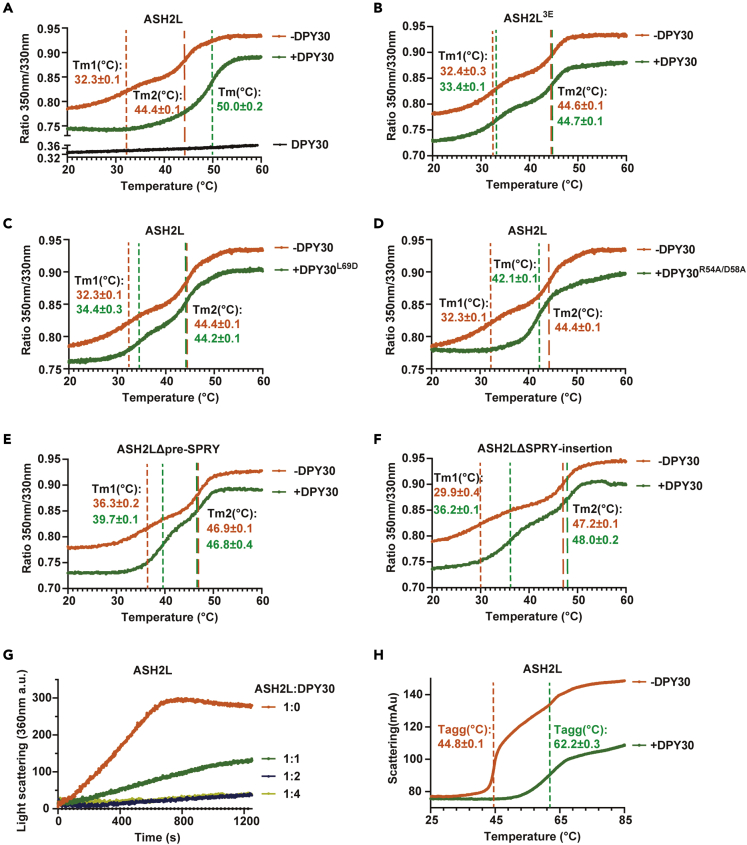
DPY30 stabilizes ASH2L (A) Unfolding curves for ASH2L (orange), ASH2L + DPY30 (mixing ASH2L and DPY30 at a molar ratio of 1:2) (green) and DPY30 (dark) obtained by nanoDSF. The Yaxis is the ratio of intrinsic fluorescence emission 350 nm/330 nm. The dashed lines indicate the melting temperature (T_m_). T_m_ values are shown as the mean ± SD (n = 3). (B) Unfolding curves for ASH2L^3E^ (orange) and ASH2L^3E^+DPY30 (green). 3E: L513E/L517E/V520E. (C) Unfolding curves for ASH2L (orange) and ASH2L + DPY30^L69D^ (green). (D) Unfolding curves for ASH2L (orange) and ASH2L + DPY30^R54A/D58A^ (green). (E) Unfolding curves for ASH2L_Δpre-SPRY_ (orange) and ASH2L_Δpre-SPRY_ + DPY30 (green). (F) Unfolding curves for ASH2L_ΔSPRY-insertion_ (orange) and ASH2L_ΔSPRY-insertion_ + DPY30 (green). (G) The ASH2L aggregation curves monitored by light scattering at 360 nm. (H) Temperature-dependent aggregation curves for ASH2L (orange) and ASH2L + DPY30 (green) obtained by nanoDSF. The dotted lines indicate the temperature of aggregation (Tagg). Data are shown as the mean ± SD (n = 3).

We then explored how the ASH2L-DPY30 interaction affects the structures and functions of ASH2L and the MLL1 complex. The structural prediction indicated that DPY30 could restrain the turbulent motion between two ASH2L lobes, leading to stabilized ASH2L with a relatively fixed conformation (Figure S4F). We wondered whether the structural stabilization of ASH2L by DPY30 could lead to the functional improvement of ASH2L. We first compared the thermostability of ASH2L or the ASH2L-DPY30 complex by nano differential scanning fluorimetry (nanoDSF), which monitors the intrinsic tryptophan fluorescence of proteins. Because DPY30 does not contain any tryptophan, the fluorescence signals reflect the folding status of ASH2L. The unfolding curves clearly showed that ASH2L exhibited a polyphasic unfolding transition with the first melting temperature (Tm1) at 32.3°C and the second melting temperature (Tm2) at 44.4°C, consistent with the multiple independent structural domains in ASH2L. The presence of DPY30 yielded a cooperative unfolding transition with one melting temperature at 50.0°C (Figure 4A), indicating that the ASH2L-DPY30 complex exhibits a compact fold with a much higher thermostability than ASH2L. Disrupting the ASH2L-DPY30 interaction by introducing ASH2L^3E^ or DPY30^L69D^ abolished the DPY30-dependent Tm increase (Figures 4B and 4C). The DPY30^R54A/D58A^mutant only slightly increased the Tm of ASH2L (Figure 4D). Deletion of pre-SPRY or SPRY-insertion of ASH2L, which impaired the ASH2L-DPY30 interaction, also severely destabilized the ASH2L-DPY30 complex with slightly increased Tm1 and unchanged Tm2 compared to ASH2L alone (Figures 4E and 4F). These results highlight the essential role of DPY30 in improving the thermostability of ASH2L.

We then checked the ability of DPY30 to prevent ASH2L aggregation. We found that the addition of increasing amounts of DPY30 substantially reduced ASH2L aggregation at 37°C as monitored by light scattering (Figure 4G). A stoichiometric quantity of DPY30 (DPY30:ASH2L = 2:1) effectively inhibited aggregation, and extra DPY30 did not further suppress the aggregation of ASH2L Figure 4G). The temperature-dependent protein aggregation curves measured by nanoDSF further confirmed that DPY30 substantially increased the T_agg_ (temperature of aggregation) of ASH2L from 44.8°C to 62.2°C Figure 4H). Collectively, DPY30 can decrease the internal structural flexibility of ASH2L to maintain a compact conformation of ASH2L and stabilize ASH2L with increased thermostability and a reduced aggregation tendency.

### DPY30 promotes the assembly of the MLL1 complex

We speculate that DPY30-dependent ASH2L stabilization may enhance ASH2L interaction with other proteins and facilitate the formation of a more stable MLL1 complex with increased HKMT activity. The crosslinking-MS data partially supported this speculation. DPY30 induced a substantial enrichment of the intramolecular crosslinks in ASH2L and intermolecular crosslinks in MLL1-ASH2L and RBBP5-ASH2L pairs (Figure 2D), indicating an enhanced internal interaction network in MWRA on DPY30 binding.

**Figure 5:**
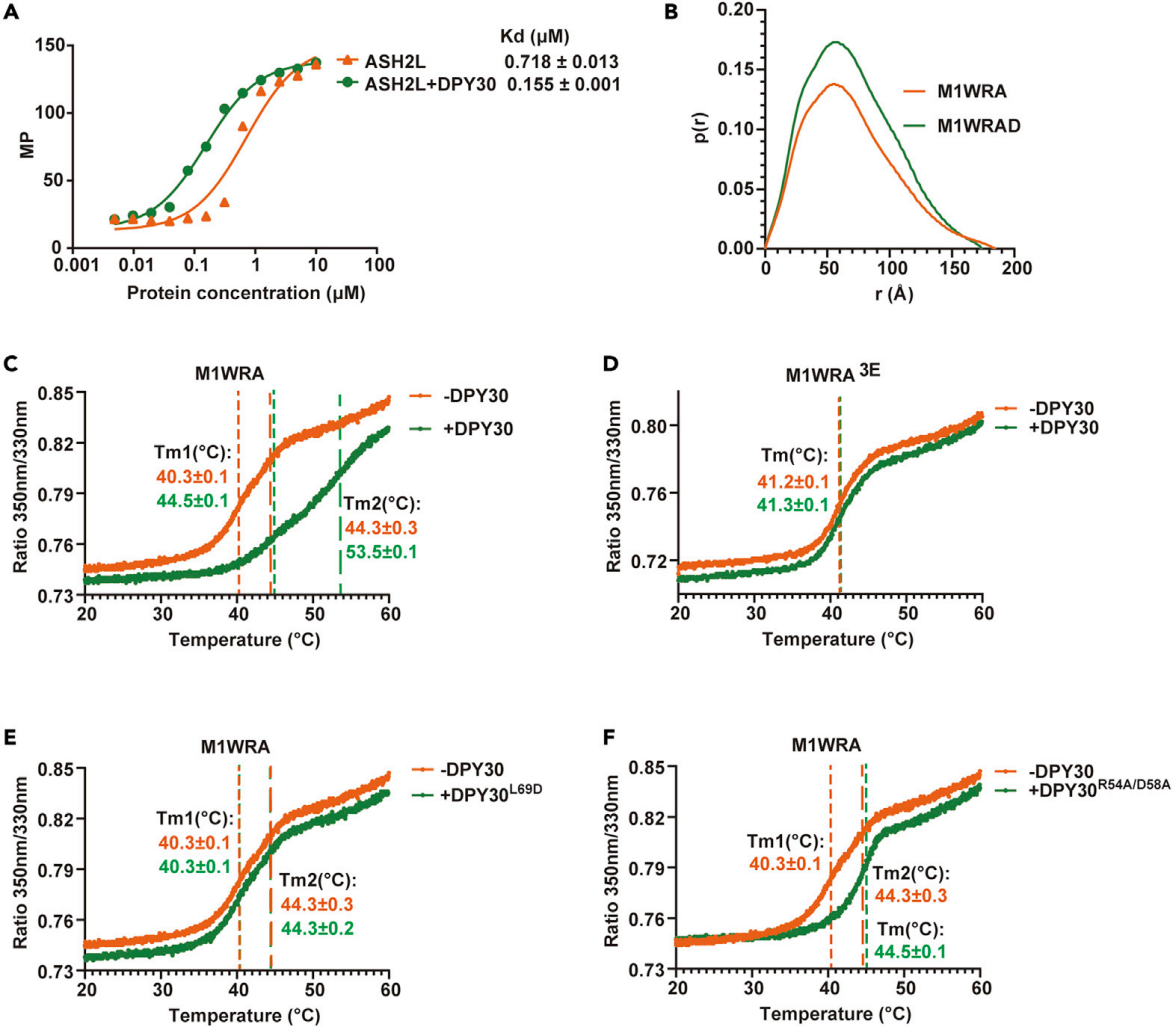
DPY30 promotes the assembly of the MLL1 complex (A) Fluorescence polarization assays showed that DPY30 remarkably enhanced the binding affinity between ASH2L and RBBP5_330-363_ (FAM-labeled at the C-terminus). Data are shown as the mean ± SD from three independent measurements. (B) Overlay of the P(*r*) distributions of M1WRA (orange) and M1WRAD (green) from SAXS assays. The *d*_*max*_ values for M1WRA and M1WRAD are 185 Å and 175 Å, respectively. (C) Unfolding curves of M1WRA (orange) and M1WRA + D (green) obtained by nanoDSF. The Yaxis is the ratio of intrinsic fluorescence emission 350 nm/330 nm. The dashed lines indicate the melting temperature (T_m_). T_m_ values are shown as the mean ± SD from three independent measurements. (D) Unfolding curves of M1WRA (orange) and M1WRA + D (green) reconstituted with ASH2L^3E^ that disrupted the interaction with DPY30. (E) Unfolding curves for M1WRA (orange) and M1WRA + DPY30^L69D^ (green). (F) Unfolding curves for M1WRA (orange) and M1WRA + DPY30^R54A/D58A^ (green). See also Figure S7 and Table S2.

To provide additional evidence that DPY30 binding may allosterically regulate ASH2L’s interaction with other proteins, we performed fluorescence polarization (FP) assays to test whether DPY30 modulates the ASH2L-RBBP5 interaction. For FP assays, we used a fluorescence-labeled RBBP5_AS-ABM_ (residues 330-363), a minimal RBBP5 fragment required for ASH2L binding (Li et al., 2016). Our FP assays showed that RBBP5_AS-ABM_ binds to free ASH2L with a dissociation constant (K_d_) of 0.72 μM, but it binds to DPY30-bound ASH2L with a K_d_ of 0.16 μM, a 4-fold increase in binding affinity (Figure 5A). These results suggest that DPY30 binding to ASH2L confers positive cooperativity for the ASH2L-RBBP5 interaction.

DPY30-dependent enhancement of internal interactions in the MLL1 complex might result in a more compact M1WRAD complex than M1WRA. To check whether DPY30 contributes to the assembly of the MLL1 complex, we utilized small-angle X-ray scattering (SAXS) to characterize the conformation of the M1WRA in the presence or absence of DPY30. The Guinier regions of the scattering curves were linear at low *q* (range of momentum transfer), indicating that the samples were not aggregated (Figures S7A and S7B). The SAXS data were used to calculate the maximum particle dimension (*d*_max_) and the radius of gyration (*R*_g_). Although the molecular weight of the M1WRAD complex (203.3 kDa) is 12.4% larger than M1WRA (180.8 kDa), the M1WRAD complex has a similar *Rg* as M1WRA and a smaller *d*_*max*_ (175 Å) than that of M1WRA (185 Å) (Figures 5B and Table S2), suggesting that DPY30 promotes the compaction of the MLL1 complex.

DPY30-induced compaction of the MLL1 complex might ensure a more stable DPY30-containing MLL1 complex. Indeed, nanoDSF analyses showed that DPY30 could elevate the Tm1 and Tm2 of the MLL1 complex from 40.3°C to 44.5°C and 44.3°C–53.5°C, respectively Figure 5C), but could not increase the Tm of the MLL1 complex reconstituted with DPY30-binding-deficient ASH2L (ASH2L^3E^) (Figure 5D). Moreover, DPY30 L69D and R54A/D58A mutations impaired the stabilization ability of DPY30 because these DPY30 mutants only mildly increased the Tm of the MLL1 complex (Figures 5E and 5F). These results indicate that DPY30-dependent ASH2L stabilization promotes the assembly and stability of the MLL1 complex. We reason that this DPY30-induced structural stabilization and compaction of the MLL1 complex may account for the mild nucleosome-independent activity stimulation by DPY30 on non-nucleosome substrates, including histone octamers, H3-H4 tetramers, and H3 peptides (Figures 1B and 1D).

### The DPY30-ASH2L complex provides additional anchors on nucleosomes

**Figure 6:**
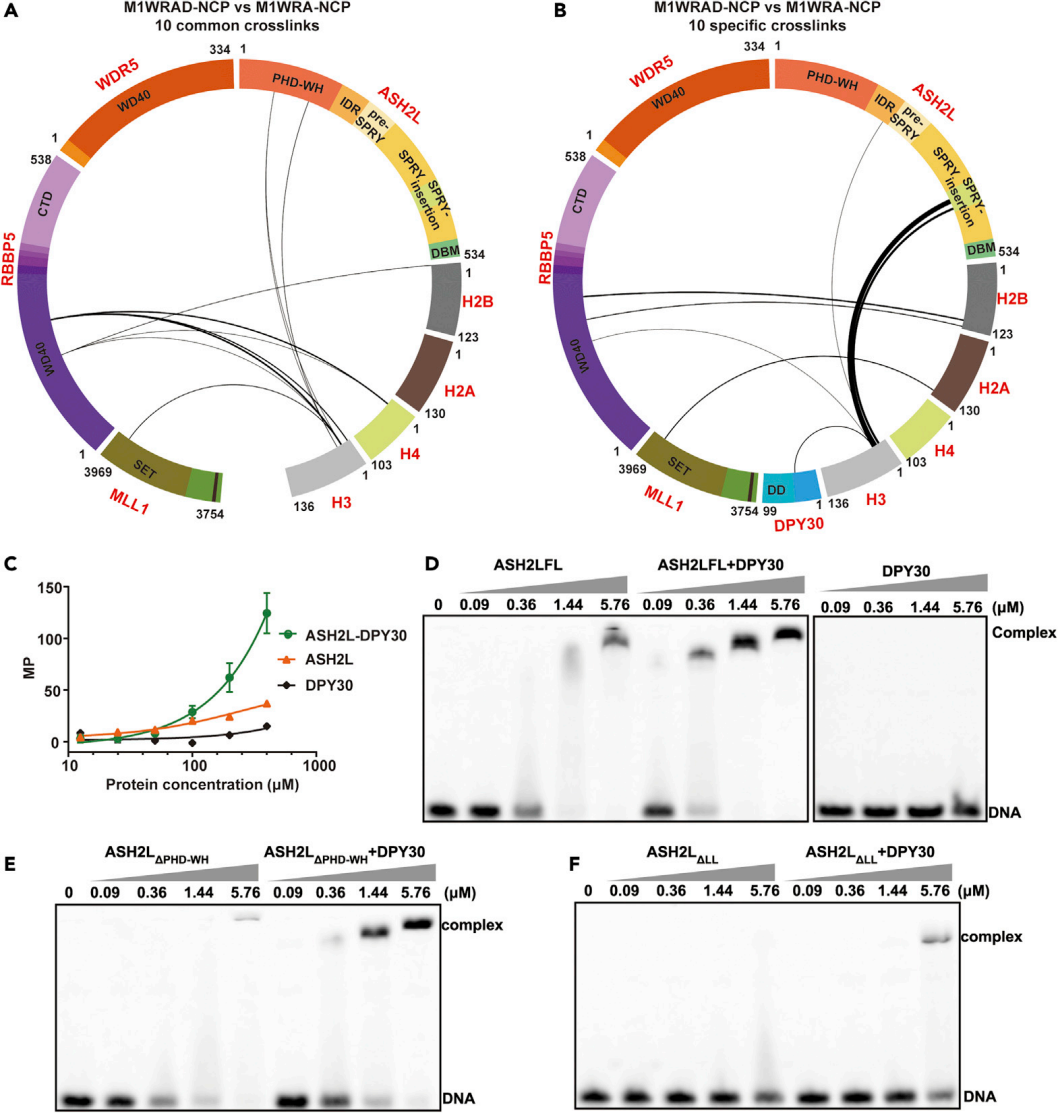
The DPY30-ASH2L complex provides additional anchors on nucleosomes (A) Circular plot of the shared crosslinks detected both in M1WRA-NCP and M1WRAD-NCP. For clarity, only the intersubunit crosslinks between M1WRA and NCP are shown. The thickness of the line is correlated with the spectrum number of the crosslinked peptide detected. (B) Circular plot of specific crosslinks detected in M1WRAD-NCP but not in M1WRA-NCP. Only the intersubunit crosslinks between M1WRAD and NCP are shown. (C) Fluorescence polarization assays showed that DPY30 enhanced the binding affinity between ASH2L and the FAM-labeled H3_1-36_ peptide. The K_d_ was not determined because of unsaturated binding even at the highest ASH2L-DPY30 concentration tested. Mean ± SD (n = 3) are shown. (D) The electrophoretic mobility shift assay (EMSA) showed that DPY30 increased the interaction between ASH2L and the FAM-labeled 25 bp dsDNA. The concentrations of ASH2L are indicated on the top, and dsDNA was 40 nM. (E) EMSA showed that ASH2L_ΔPHD-WH_ had a decreased DNA binding ability, but the ASH2L_ΔPHD-WH_–DPY30 complex had a similar DNA binding ability as wild type ASH2L-DPY30. F. EMSA showed that ASH2L_ΔLL_ failed to bind DNA and that the ASH2L_ΔLL_–DPY30 complex had severely decreased DNA binding ability. ΔLL indicates the deletion of ASH2L IDR, pre-SPRY, and SPRY-insertion. See also Figure S8 and Table S3.

Although DPY30-induced compaction of the MLL1 complex could explain the nucleosome-independent activity stimulation by DPY30, an additional mechanism must exist that determines the nucleosome-specific activity boosted by DPY30. To probe how DPY30 affects the M1WRA-NCP interaction, we performed crosslinking mass spectrometry analyses of the M1WRAD-NCP and M1WRA-NCP complexes, aiming to identify potential M1WRAD-NCP interfaces induced by DPY30. There were 102 crosslinked peptides identified in the M1WRA-NCP sample, and 10 of them were between M1WRA and NCP histones (Figures S8A, S8B and S8D and Data S2). For comparison, 20 out of 148 crosslinked peptides were identified between M1WRAD and NCP histones (Figures S8A, S8C and S8E and Data S2). The 10 crosslinked peptides between M1WRA and NCP histones identified in M1WRA-NCP were all found in M1WRAD-NCP (Figure 6A). In the presence of DPY30, there are 10 specific crosslinks between M1WRAD and NCP (Figure 6B), assumably providing additional anchors for M1WRAD on nucleosomes.

Notably, the N-terminal tail of H3 was extensively crosslinked to the SPRY-insertion and IDR elusively found in M1WRAD-NCP, indicating a potential direct interaction between ASH2L and the H3 tail in the presence of DPY30 Figure 6B). To provide direct evidence that DPY30 may enhance ASH2L’s interaction with the H3 tail, we used a fluorescence polarization assay to characterize the ASH2L interaction with a fluorescent H3_1-36_ peptide. FP assays showed that ASH2L or DPY30 alone had a negligible H3-binding ability, but the ASH2L-DPY30 complex had an appreciable interaction with the H3 tail Figure 6C), suggesting that DPY30-stabilized ASH2L could interact with the H3 tail.

In addition to the ASH2L-H3 interfaces, DPY30-stabilized ASH2L may achieve the ability to bind DNA, as indicated by the previous M1WRAD-NCP structure showing the close proximity between ASH2L and nucleosomal DNA (Park et al., 2019; Xue et al., 2019). Because our previous studies demonstrated that ASH2L had DNA-binding activity (Chen et al., 2011), we wondered whether DPY30 might modulate ASH2L’s DNA-binding activity. The electrophoretic mobility shift assay (EMSA) confirmed that apo ASH2L had DNA-binding activity but mostly formed protein-DNA aggregates not migrating into the gel, as judged by the density of the shifted band (Figure 6D). Although DPY30 itself does not have any DNA-binding activity, the inclusion of DPY30 not only increased the binding affinity between ASH2L and DNA but also facilitated the formation of a soluble protein-DNA complex running as a sharp band on EMSA gels Figure 6D).

Previous studies have shown that the PHD-WH domain of ASH2L can bind DNA (Chen et al., 2011; Sarvan et al., 2011). We found that the deletion of PHD-WH (ASH2L_ΔPHD-WH_) indeed decreased the ASH2L-DNA association, but ASH2L_ΔPHD-WH_ still responded to DPY30. DPY30 greatly increased the binding affinity between ASH2L_ΔPHD-WH_ and DNA (Figure 6E), consistent with the observation that the deletion of PHD-WH had a marginal effect on the HKMT activity on NCP (Figure 2B). The ASH2L IDR, pre-SPRY, and SPRY-insertion motifs are more critical for DNA-binding activity of ASH2L. The deletion of these motifs (ASH2L_ΔLL_) completely abolished the DNA-binding ability of ASH2L and severely disrupted the DPY30-dependent DNA-binding activity of ASH2L (Figure 6F). Thus, we conclude that the presence of DPY30 makes ASH2L competent for DNA binding through ASH2L IDR, pre-SPRY, and SPRY-insertion motifs. Taken together, these results support the notion that DPY30-stabilized ASH2L acquires additional interaction interfaces with histones and DNA, which may reduce the dynamics of the MLL1 complex on NCP and ensure the correct priming of the H3 substrate to boost the enzymatic activity of the MLL1 complex on nucleosomes.

### The conserved role of DPY30 in stimulating HKMT activity of MLL-family complexes

**Figure 7:**
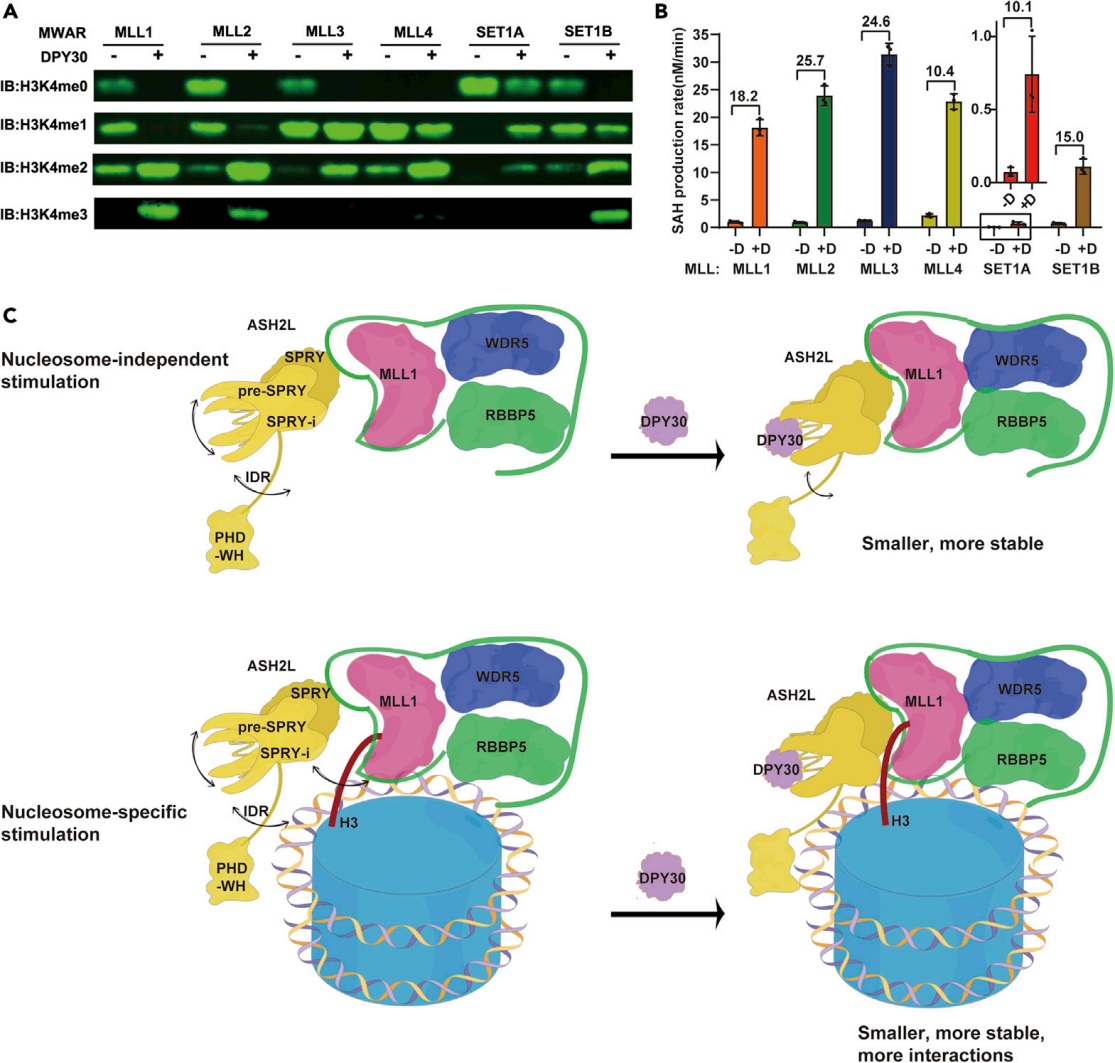
The conserved role of DPY30 in stimulating HKMT activity of MLL-family complexes (A) Methyltransferase activities of different MLL complexes with and without DPY30 (MWRA and MWRA + D) on NCP substrates were characterized by western blot. (B) Methylation rates of different MWRA and MWRA + D on NCP measured by the MTase-Glo Methyltransferase Assay kit. Fold changes in methylation rates between MWRAD and MWRA are indicated on the top. Data are shown as the mean ± SD (n = 3). The SET1A complex has relatively low activity, so an inset shows an enlarged graph for comparison of the methylation rates of the SET1A complex. (C) The model for activity regulation of MLL complexes by DPY30. DPY30 stimulates the HKMT activity of MLL complexes through a nucleosome-independent mechanism and a nucleosome-specific mechanism.

To confirm whether DPY30 is a universal stimulator for all MLL family methyltransferase complexes, we used a western blot-based activity assay to test the HKMT activities of other MLL family complexes in the presence and absence of DPY30 on NCP substrates. Similar to the observation in the MLL1 complex, DPY30 enhanced the HKMT activity of other MLL family complexes on NCP to different extents (Figure 7A). We then measured the reaction rates of different MLL family complexes by using the MTase-Glo Methyltransferase Assay kit. The MLL complexes with DPY30 possessed 10- to 25-fold higher methyltransferase activity than the corresponding complexes without DPY30 Figure 7B). These results demonstrate that the DPY30-dependent activity-stimulation mechanism derived from studies of the MLL1 complex can also be applied to other MLL family methyltransferases.

## Discussion

DPY30 is the smallest subunit (99 amino acids) in the MLL complex and associates peripherally with the MLL complex through its interaction with ASH2L (Patel et al., 2009). Although DPY30 plays an essential role in maintaining H3K4 methylation levels *in vivo* (Jiang et al., 2011; Yang et al., 2014, 2016), its biochemical role in maintaining the HKMT activity of the MLL complex has been underestimated. Here, we demonstrate that DPY30 plays a crucial role in the activity regulation of the MLL complex through two complementary mechanisms: a nucleosome-independent mechanism and a nucleosome-specific mechanism.

First, DPY30 improves the stability of ASH2L by interacting with much broader interfaces of ASH2L than previously characterized. The stabilized ASH2L gains multiple functional improvements, including increased thermal stability, less aggregation, and enhanced interaction with RBBP5. All these DPY30-dependent properties collectively contribute to the assembly of a compact and stable MLL complex with enhanced HKMT activity Figure 7C). This DPY30-dependent compaction and stabilization of the MLL complex could explain the previously ignored nucleosome-independent activity stimulation by DPY30, as observed on histone octamer, H3-H4 tetramer, and H3 peptide (Figure 1B).

Second, DPY30 significantly enhances the HKMT activity of the MLL complex on nucleosomes (Kwon et al., 2020; Lee et al., 2021) (Figure 1C). This nucleosome-specific activity enhancement relies on the newly generated interfaces between DPY30-stabilized ASH2L and nucleosomes. The extra interfaces of ASH2L-H3 and ASH2L-DNA may ensure a relatively fixed configuration of the MLL complex on nucleosomes, thereby boosting the HKMT activity of the MLL complex (Figure 7C). It should be noted that the major role of these newly generated interfaces is not to increase the binding affinity between the MLL complex and nucleosomes. Our kinetic analyses showed that DPY30 decreased the *K*_m_ by only 1.5-fold (Figure 1C). Thus, the primary roles of these newly generated ASH2L-nucleosome interfaces are to prime the MLL1 complex in a correct orientation on nucleosomes and facilitate H3 alignment into the active pocket of the SET domain to catalyze H3K4 methylation more efficiently. This can explain why DPY30 causes more of a change in *k*_cat_ rather than a *K*_m_ change (Figure 1C).

A recent report from the Dou laboratory concluded that DPY30 enhanced the activity of the MLL1 complex on nucleosomes by restricting the rotational dynamics of the MLL1 complex on NCP (Lee et al., 2021). Their work and our present study complement each other to reveal how ASH2L and DPY30 interact to affect the conformation of the MLL complex on nucleosomes. Notwithstanding the similar conclusions we both held, our studies reveal some new aspects of the ASH2L-DPY30 interaction. For example, the study from the Dou lab only addressed the nucleosome-specific activation mechanism (Lee et al., 2021), but our study also reveals the nucleosome-independent mechanism, explaining the activity stimulation by DPY30 on broad-spectrum substrates. Moreover, our study provides direct biochemical evidence that DPY30 enhances ASH2L’s interactions with the H3 tail and DNA (Figure 6), explaining why DPY30 reduces the rotation dynamics of the MLL1 complex on NCP.

In addition, our study provides an alternative explanation for how DPY30 affects the structures and functions of ASH2L. Dou’s work emphasized the importance of ASH2L IDRs and proposed that the major role of DPY30 was to induce the conformational change of ASH2L IDRs (Lee et al., 2021). Here, we show that the so-called ASH2L IDRs in the previous publication (Lee et al., 2021), especially the pre-SPRY and SPRY-insertion motifs, have well-defined structural features and are not obviously altered by DPY30 binding (Figures S4 and S5). The major role of DPY30 is not to induce the conformational change of ASH2L but to increase the stability of ASH2L and allosterically enhance ASH2L-mediated interactions (including interactions with RBBP5, H3, and DNA). DPY30-dependent enhancement of the internal interaction networks thus facilitates the formation of a compact MLL core complex and reduces the conformational flexibility of the MLL complex on nucleosomes.

The functions of DPY30 in increasing ASH2L thermostability, preventing ASH2L aggregation, and enhancing the ASH2L-dependent methyltransferase activity of the MLL1 complex are analogous to protein chaperones in regulating the stabilities and activities of client proteins. We propose that DPY30 functions as an ASH2L-specific chaperone to stabilize ASH2L. Whether DPY30 functions as a general chaperone remains to be determined. Notwithstanding, DPY30 is the peripheral protein of the MLL complex, and the dissociation of DPY30 does not lead to the disassembly of the MLL complex (Patel et al., 2009) but rather “turns off” the HKMT activity of the MLL1 complex. In certain circumstances, the MLL complex binds to the target chromatin regions in the priming state (without DPY30, low activity) and waits for the signal to quickly switch to the activation state (with DPY30 binding, high activity) to “turn on” HKMT activity. Therefore, DPY30 may serve as a delicate on/off switch for MLL-family complexes to precisely regulate the HKMT activity.

Owing to the critical role of DPY30 in maintaining H3K4 methylation, abnormal expression of DPY30 can lead to the initiation and progression of human diseases. Extensive studies have reported that DPY30 is overexpressed in many types of cancers, accompanied by increased H3K4me3 modification in cancer cells (Dixit et al., 2022; Gu et al., 2021; He et al., 2019; Hong et al., 2020; Lee et al., 2015; Shah et al., 2019; Yang et al., 2018; Zhang et al., 2018). Overexpression of DPY30 promotes proliferation, migration, and invasion of tumor cells (Lee et al., 2015; Yang et al., 2018; Zhang et al., 2018). Thus, we hypothesize that DPY30 may act as an “accelerator” for the MLL complex to drive aberrant gene expression in cancer cells. The DPY30-ASH2L interface might be a therapeutic target for cancer treatment. As the first proof-of-concept for targeting the DPY30-ASH2L interaction, a peptide derived from ASH2L_DBM_ (residues 510-529) decreased the global H3K4me3 and modestly inhibited the growth of MLL-rearranged leukemia (Shah et al., 2019), demonstrating the feasibility of targeting the DPY30-ASH2L interaction for cancer treatment. The newly identified DPY30-ASH2L interface and ASH2L-NCP interface revealed in the current study provide a foundation for designing or screening inhibitors with high potency and specificity to target the “DPY30-ASH2L-MLL-NCP” axis, hopefully contributing to the discovery of new therapeutic drugs for certain cancers.

### Limitation of the study

Here we used the ColabFold powered by AlphaFold2 to predict the structure of the ASH2L-DPY30 complex. Although the predicted structural model looks plausible and has been supported by mutagenesis studies, the exact structure of the ASH2L-DPY30 complex still awaits experimental determination by X-ray crystallography or other structural methodologies, which will reveal more detailed interface information between ASH2L and DPY30. The structural information will provide a foundation for the rational design of small molecules or peptide mimics to inhibit the ASH2L-DPY30 interaction for potential therapeutic usage.

Our biochemical data suggest that multiple regions of ASH2L and DPY30 are essential for maintaining the HKMT activity of MLL complexes on NCP. Unfortunately, currently available cryo-EM structures of MWRAD-NCP had the lowest resolution (8-12 Å) in the ASH2L-DPY30 region, preventing us from building a reliable model for ASH2L-DPY30 on NCP. Moreover, the conformational change of ASH2L-DPY30 on binding with NCP is expected, especially in the highly flexible ASH2L IDR region (aa 178-229). Therefore, the high-resolution cryo-EM structure of MWRAD-NCP is required to dissect how ASH2L-DPY30 impacts the methylation ability of the MLL complexes on nucleosomes.

## STAR★Methods

### Key resources table

**Table:** 

REAGENT or RESOURCE	SOURCE	IDENTIFIER
**Antibodies**		

Rabbit monoclonal anti-H3 total	Cell Signaling Technology	Cat# 4499S; RRID:AB_10544537
Mouse monoclonal anti-H3K4me0	Active Motif	Cat# 39763; RRID:AB_2650522
Rabbit polyclonal anti-H3K4me1	Active Motif	Cat# 39297; RRID:AB_2615075
Rabbit polyclonal anti-H3K4me2	Millipore	Cat# 07-030; RRID:AB_310342
Rabbit monoclonal anti-H3K4me3	Cell Signaling Technology	Cat# 9751S; RRID:AB_2616028
IRDye 800CW goat anti-rabbit IgG	LI-COR Biosciences	Cat# 926-32211, RRID:AB_621843
IRDye 680RD goat anti-mouse IgG	LI-COR Biosciences	Cat# 926-68070, RRID:AB_10956588

**Bacterial and virus strains**		

Transetta (DE3)	TransGen Biotech	Cat# CD801-02
BL21 (DE3)	TransGen Biotech	Cat# CD601-02

**Chemicals, peptides, and recombinant proteins**		

FastPfu Fly DNA polymerase	TransGen Biotech	Cat# AP231-13
ClonExpress II OneStep Cloning Kit	Vazyme	Cat# C112-01
SPARKeasy Superpure Mini Plasmid Kit	Sparkjade	Cat# AD0102-C
MTase-Glo Methyltransferase Assay Kit	Promega	Cat# V7602
Prometheus NT.48 Series nanoDSF Grade Standard Capillaries	NanoTemper	Cat# PR-C002
Ni-NTA Agarose	QIAGEN	Cat# 30230
Glutathione Sepharose 4B	GE Healthcare	Cat# 17-0756-05
IPTG	AMRESCO	Cat# 0487
Reduced glutathione	AMRESCO	Cat# 0399
PMSF	Sigma-Aldrich	Cat# 329-98-6
AdoMet	Sigma-Aldrich	Cat# A7007
AdoHcy	Sigma-Aldrich	Cat# A9384
Disuccinimidyl suberate (DSS)	ThermoFisher	Cat# 21655
H3_1-9_ peptide (ARTKQTARY)	Lifetine	N/A
FAM-labeled H3_1-36_ peptide (ARTKQTARKSTGGKAPRKQLA TKAARKSAPATGGVK-FAM)	Lifetine	N/A
FAM-labeled RBBP5_330-363_ peptide (SAFAPDFKELDENVEYEERESEFDIE DEDKSEPEK-FAM)	Lifetine	N/A

**Deposited data**		

SAXS data of MLL1-WDR5-RBBP5-ASH2L complex	This paper	SASDDB: SASDPT3
SAXS data of MLL1-WDR5-RBBP5-ASH2L-DPY30 complex	This paper	SASDDB: SASDPU3
Raw SDS-PAGE gels and western blot images	Mendeley Data	http://dx.doi.org/10.17632/s9g2mr6ytx.1

**Oligonucleotides**		

FAM-labeled dsDNA (FAM-TCTCTA GAGTCGACCTGCAGGCATG)	Genscript	N/A

**Recombinant DNA**		

pGEX6p1-GST-MLL1 3754-3969	This paper	N/A
pGEX6p1-GST-MLL2 2508-2715	This paper	N/A
pGEX6p1-GST-MLL3 4700-4911	This paper	N/A
pGEX6p1-GST-MLL4 5335-5537	This paper	N/A
pGEX6p1-GST-SET1A 1491-1707	This paper	N/A
pGEX6p1-GST-SET1B 1706-1923	This paper	N/A
pGEX6p1-GST-DPY30 FL	This paper	N/A
pGEX6p1-GST-ASH2L FL	This paper	N/A
pET-Sumo-WDR5 FL	This paper	N/A
pET-Sumo-RBBP5 FL	This paper	N/A
pET-Sumo-ASH2L FL	This paper	N/A
pET-Sumo-DPY30 FL	This paper	N/A
pET-Sumo-ASH2L Δ1-177	This paper	N/A
pET-Sumo-ASH2L Δ1-230	This paper	N/A
pET-Sumo-ASH2L Δ178-229	This paper	N/A
pET-Sumo-ASH2L Δ230-285	This paper	N/A
pET-Sumo-ASH2L Δ400-440	This paper	N/A
pET-Sumo-ASH2L Δ178-285Δ400-440	This paper	N/A
pET-Sumo-ASH2L L513E/L517E/V520E	This paper	N/A
pET-Sumo-ASH2L K413A	This paper	N/A
pET-Sumo-ASH2L R280A	This paper	N/A
pET-Sumo-ASH2L D515A/Y518A/H519A	This paper	N/A
pET-Sumo-DPY30 Δ1-44	This paper	N/A
pET-Sumo-DPY30 Δ45-99	This paper	N/A
pET-Sumo-DPY30 L69D	This paper	N/A
pET-Sumo-DPY30 R54A/D58A	This paper	N/A
pET4D-xlH2A FL	This paper	N/A
pET4D-xlH2B FL	This paper	N/A
pET4D-xlH3 FL	This paper	N/A
pET4D-xlH4 FL	This paper	N/A
pUC19-Widom 601 DNA	This paper	N/A

**Software and algorithms**		

Image Studio 5.2.5	LI-COR Biosciences	5.2.5 https://www.licor.com/bio/image-studio/
GraphPad Prism 8.0	Graphpad Software, San diego, CA	8.0 https://www.graphpad.com/scientific-software/prism/
Charticulator	Microsoft	https://charticulator.com/app/index.html
PR. ThermControl	Nano Temper	https://nanotempertech.com/zh_cn/prometheus-pr-thermcontrol-software/
ColabFold	Mirdita et al., 2022	https://colabfold.mmseqs.com/
PyMOL	Schrodinger, LLC.	https://pymol.org
GROMACS	Kutzner et al., 2015	https://manual.gromacs.org/current/download.html
CHARMM36	Huang and MacKerell, 2013	http://mackerell.umaryland.edu/charmm_ff.shtml
VMD 1.9.1	Humphrey et al., 1996	https://www.ks.uiuc.edu/Research/vmd/vmd-1.9.1/
Origin 9	OriginLab, Northampton, MA	https://www.originlab.com/
BioXTAS RAW	Hopkins et al., 2017	https://sourceforge.net/projects/bioxtasraw/
ATSAS 3.0.3	Franke et al., 2017	https://www.embl-hamburg.de/biosaxs/download.html

### Resource availability

#### Lead contact

Further information and requests for resources and reagents should be directed to and will be fulfilled by the lead contact, Yong Chen (yongchen@sibcb.ac.cn).

#### Material availability

This study did not generate new unique reagents. Any additional resources in this paper are available from the lead contact on request.

### Experimental model and subject details

We used *Escherichia coli* Transetta (DE3) cells for protein production. The cells were cultured in LB media at 37°C until OD600 reached 0.6, and then induced by 0.1 mM IPTG at 18°C for 16–18 h.

### Method details

#### Plasmid construction

Each of the six human MLL family proteins used in this study only contains the WIN motif and the SET domain. We used the following constructs: MLL1 (Q03164) 3754-3969, MLL2 (Q9UMN6) 2508-2715, MLL3 (Q8NEZ4) 4700-4911, MLL4 (O14686) 5335-5537, SET1A (O15047) 1491-1707, and SET1B (Q9UPS6) 1706-1923. MLL1, MLL2, MLL3, MLL4, SET1A, SET1B, ASH2L (Q9UBL3-3), DPY30 (Q9C005), and their truncations or mutants were individually inserted into the pGEX6p-1 vector with a GST tag at the N-terminus. WDR5 (P61964), RBBP5 (Q15291), ASH2L (Q9UBL3-3), DPY30 (Q9C005), and their truncations or mutants were cloned into a modified pET28b vector with a 6×His-sumo tag at the N-terminus. These plasmids were extracted using the SPARKeasy Superpure Mini Plasmid Kit (Sparkjade Science, China) and further validated by Sanger sequencing.

#### Protein expression and purification

*Escherichia coli* Transetta (DE3) cells (TransGen Biotech, China) bearing expression plasmids were induced with 0.2 mM isopropyl β-D-1-thiogalactopyranoside (IPTG) at 16°C for 16-18 h. We added 20 μM ZnSO_4_ to the media for MLL expression. After induction, cells were harvested by centrifugation and resuspended in lysis buffer (50 mM Tris-HCl pH 8.0, 400 mM NaCl, 10% glycerol, 2 mM 2-mercaptoethanol, and protease inhibitor cocktail). Cells were then lysed by ultrasonication, followed by centrifugation at 18000 rpm for 50 min to remove cell debris. The supernatant was incubated with Ni-NTA agarose beads (Qiagen, USA) for 6xHis-sumo-tagged proteins or glutathione Sepharose 4B beads (GE Healthcare, USA) for GST-tagged proteins at 4°C. The beads were then washed with lysis buffer, and the flow-through was monitored by the Bradford protein assay. Tag-free proteins were eluted by on-column digestion with ULP1 protease for His-sumo tagged proteins or 3C protease for GST tagged proteins. GST-tagged proteins used in GST pull-down assays were directly eluted in elution buffer (50 mM Tris-HCl pH 8.0, 400 mM NaCl, 10% glycerol, and 15 mM reduced glutathione) without protease digestion. Proteins were further purified by size-exclusion chromatography on a Hiload Superdex 75 or Hiload Superdex 200 column (GE Healthcare, USA) equilibrated with 25 mM Tris-HCl, pH 8.0, 150 mM NaCl. The buffer for size-exclusion chromatography to purify MLL family proteins contained 25 mM Tris-HCl pH 8.0, 300 mM NaCl, and 10% glycerol.

After purifying all components separately, all MLL-WRA complexes were obtained by mixing MLL protein, WDR5, RBBP5, and ASH2L at a molar ratio of 1.8:1.2:1:1. The MLL-WRAD complex was obtained by mixing MLL, WDR5, RBBP5, ASH2L, and DPY30 at a molar ratio of 1.8:1.2:1:1:4. After incubation on ice for 1 h, the assembled MLL complexes were separated from free individual components through a Superdex 200 Increase (10/300 GL) column (GE Healthcare, USA) in buffer containing 25 mM HEPES pH 7.4, 150 mM NaCl, and 2 mM DTT. The fractions containing the stoichiometric complex identified by SDS-PAGE were concentrated, flash-frozen, and stored at −80°C.

There are two kinds of complexes in our studies. “MWRAD” represents the preassembled MWRAD complex purified by size-exclusion chromatography, which was used in crosslinking-MS and SAXS experiments that prefer homogeneous samples. “MWRA + D” represents the mixing of DPY30 and the preassembled MWRA complex, which is incubated on ice for 30 min before conducting subsequent experiments, including methyltransferase assays and nanoDSF assays.

#### Nucleosome reconstitution

Nucleosome reconstitution was prepared as previously described (Dyer et al., 2004). In brief, the *Xenopus laevis* histones H2A, H2B, H3, and H4 were expressed separately in *E. coli* BL21 (DE3) and purified from inclusion bodies. Histones were purified sequentially through a Q Sepharose HP column (GE Healthcare, USA) and an SP Sepharose HP column (GE Healthcare, USA) in denaturing buffer (20 mM Tris-HCl pH 7.5, 8 M urea, 0-1 M NaCl, 1 mM EDTA, and 5 mM 2-mercaptoethanol). The purified histones were dialyzed thoroughly in distilled water containing 2 mM 2-mercaptoethanol, followed by lyophilization. Purified histones were stored at −80°C until use.

Histone octamers were assembled by mixing H2A, H2B, H3, and H4 at a molar ratio of 1.5:1.2:1:1 on ice for 1 h. H3-H4 tetramers were assembled by mixing the same molar amounts of H3 and H4. The histone octamers or H3-H4 tetramers were separated on a Hiload Superdex 200 (GE Healthcare, USA) equilibrated with 20 mM Tris-HCl pH 7.5, 2 M NaCl, 1 mM EDTA, and 5 mM 2-mercaptoethanol.

To reconstitute NCPs, we first purified 147-bp Widom-601 DNA as previously described (Dyer et al., 2004). The histone octamer and Widom-601 DNA were mixed at a molar ratio of 1:0.9, followed by dialysis in reconstitution buffer (10 mM Tris-HCl pH 7.5, 1 mM EDTA, 1 mM DTT, and 0.15–2 M KCl) over a 36-h gradient. The reconstituted NCP was further purified through Superose 6 increase 10/300 (GE Healthcare, USA), equilibrated with 10 mM Tris-HCl pH 7.5, 1 mM DTT, and 0.15 M KCl.

#### Methyltransferase assay by western blot

For the methylation reaction, 2 μM MLL1 complex (M1WRA or M1WRA + D), 2 μM NCP, and 100 μM S-adenosyl-L-methionine (SAM) were mixed in buffer containing 25 mM HEPES pH 7.4, 150 mM NaCl, and 1 mM DTT. The whole system was incubated at 30°C for 30 min. The reaction was quenched by adding trifluoroacetate (TFA) to a final concentration of 0.1%. Then, the reaction mixtures were separated by 12% SDS–PAGE and transferred onto 0.22 μM PVDF membranes (Millipore, USA). The membranes were blocked in blocking solution containing 5% nonfat powdered milk in TBS with 0.1% Tween 20 (TBST), followed by incubation at 4°C overnight with the corresponding primary antibodies (anti-H3 total, CST 4499S; anti-H3K4me0, Active Motif 39763; anti-H3K4me1, Active Motif 39297; anti-H3K4me2, Millipore 07-030; anti-H3K4me3, CST 9751S). After incubation, the membranes were washed three times with TBST and incubated at room temperature for 1 h with IRDye-labeled secondary antibodies (IRDye 680RD goat anti-mouse IgG, LI-COR P/N 926-68070; IRDye 800CW goat anti-rabbit IgG, LI-COR P/N 923-32211). Membranes were scanned by using the Odyssey CLx imaging system (LI-COR, USA) to measure fluorescence at 700 and 800 nm.

#### Methyltransferase assay by the MTase-Glo Methyltransferase Assay kit

For a methylation reaction, 40 μM SAM and 1 μM substrates (NCP, octamer, H3-H4 tetramer) or 40 μM H3_1-9_ peptide were mixed in buffer containing 25 mM HEPES pH 7.4, 100 mM NaCl, 0.1 mg/mL BSA, and 1 mM DTT. The reaction was initiated by adding 50 nM MLL1 complex (M1WRA or M1WRA + D) and then incubated at 30°C. An 8 μL reaction mixture was taken and quenched at different time points by adding 2 μL 0.5% trifluoroacetate (TFA). The methylation activities were evaluated using the MTase-Glo Methyltransferase Assay Kit (Promega, USA). In this kit, the generated cofactor product, S-adenosyl-L-homocysteine (SAH), was converted through multiple-step enzyme-coupled reactions and detected via a luciferase reaction. The luminescence signals were converted to the concentrations of SAH using a standard curve calibrated by SAH standards. The initial rate of each reaction (nM SAH/min) was determined by a linear regression fit of the data, and the standard error was calculated from triplicate reactions.

For kinetic analysis of the M1WRA complex and the M1WRA+D complex activities on NCP, 50 nM M1WRA or M1WRA+D complex was used with 62.5 nM to 1000 nM NCP and 40 μM SAM. For kinetic analysis of the M1WRA complex and the M1WRA+D complex activities on H3_1-9_, 50 nM M1WRA or M1WRA+D complex was used with 62.5 μM to 2000 μM H3_1-9_ and 500 μM SAM. The initial rates (nM SAH/min) of reactions at different substrate concentrations were measured. Steady-state kinetic parameters were determined by fitting the initial rates to the Michaelis–Menten equation using nonlinear regression in GraphPad Prism 8.0 (GraphPad, USA). The standard error was calculated from triplicate reactions.

#### Crosslinking mass spectrometry analysis

We crosslinked the M1WRA or M1WRAD complex at a final concentration of 0.6 mg/ml by disuccinimidyl suberate (DSS) with a final crosslinker concentration of 2 mM in buffer containing 25 mM HEPES, pH 7.4, 150 mM NaCl, and 1 mM DTT. To crosslink the MLL1 complex with NCP, we mixed the M1WRA or M1WRAD complex, NCP, and SAH at a molar ratio of 2:1:10 at a final concentration of 0.6 mg/m in buffer containing 25 mM HEPES pH 7.4, 150 mM NaCl, and 1 mM DTT and incubated them on ice for 30 min. We then crosslinked the M1WRA(D)-NCP-SAH complex with 2 mM DSS. After incubation at 25°C for 30 min, stop buffer (50 mM Tris-HCl, pH 7.4) was used to terminate the reaction.

The proteins were precipitated and digested by trypsin at an enzyme-to-substrate ratio of 1:50 (w/w) at 37°C for 16 h. The tryptic digested peptides were desalted and loaded on an in-house packed capillary reverse-phase C18 column (40 cm length, 100 μM ID x 360 μM OD, 1.9 μM particle size, 120 Å pore diameter) connected to an Easy LC 1200 system (Thermo Fisher Scientific, USA). The samples were analyzed with a 120 min-HPLC gradient from 6% to 35% buffer B at 300 nL/min (buffer A: 0.1% formic acid in water; buffer B: 0.1% formic acid in 80% acetonitrile). The eluted peptides were ionized and introduced into a Q Exactive mass spectrometer using a nanospray source. Survey full-scan MS spectra (from *m*/*z* 300-1800) were acquired in the Orbitrap mass analyzer with resolution r = 70,000at*m*/*z* 400. Crosslinked peptides were identified and evaluated using pLink2 software (Chen et al., 2019). Crosslinked patterns were plotted using Microsoft Charticulator.

#### GST pull-down assay

GST-fusion proteins and interacting partners were incubated with 10 μL glutathione Sepharose 4B beads (GE Healthcare, USA) for 2hat 4°C in 100 μL binding buffer (25 mM Tris-HCl pH 8.0, 300 mM NaCl, and 2 mM DTT). After the beads were washed three times with 200 μL binding buffer, the bound proteins were eluted with 25 μL elution buffer (300 mM NaCl, 25 mM Tris-HCl, pH 8.0, 20 mM reduced GSH, and 2 mM DTT). All samples were separated by 12% SDS–PAGE and stained with Coomassie brilliant blue.

#### Nano differential scanning fluorimetry assay

All nanoDSF experiments were performed on a Prometheus NT.48 system (NanoTemper Technologies, Germany) in triplicate. First, protein samples were diluted to a 1 mg/mL concentration in buffer containing 25 mM Tris-HCl, pH 8.0, and 150 mM NaCl. Next, capillaries filled with samples were placed on the loading tray and heated from 20°C to 85°C at a heating rate of 1°C/min. The fluorescence at 330 and 350 nm and the light scattering signals were recorded. The melting temperature (Tm) and the aggregation temperature (Tagg) were determined by the PR. ThermControl software (NanoTemper Technologies, Germany).

#### ASH2L aggregation assay

To monitor thermoinduced ASH2L aggregation, ASH2L was diluted into 1 mL 37°C prewarmed buffer containing 25 mM Tris-HCl pH 8.0 and 150 mM NaCl in a cuvette to a final concentration of 1.5 μM. Different ratios of DPY30 (ASH2L: DPY30 = 1:0, 1:1, 1:2, or 1:4) were then added and continuously mixed with a small magnetic stirring bar. ASH2L aggregation was monitored by light scattering at 360 nm using a Thermo LUMINA fluorescence spectrophotometer (Thermo Fisher Scientific, USA) with a Peltier temperature controller.

#### Fluorescence polarization assay

ASH2L only or ASH2L-DPY30 complexes were diluted to a series of concentrations from 432 μM to 13.5 μM in buffer containing 25 mM Tris, pH 8.0, and 150 mM NaCl. Various concentrations of proteins (30 μL) were mixed with 2.4 μL of 1.35 μM FAM-labeled H3_1-36_ peptide (ARTKQTARKSTGGKAPRKQLATKAARKSAPATGGVK-FAM) (final concentration 100 nM) and incubated on ice in the dark for 30 min. The fluorescence polarization values in 384-well black plates were measured using a Synergy *Neo* Multi-Mode Reader (Bio-Tek, USA) at an excitation wavelength of 485 nm and an emission wavelength of 528 nm. Fluorescence was quantitated with GEN 5 software (Bio-Tek, USA), and data were analyzed with GraphPad Prism 8.0 (GraphPad, USA). Notably, K_d_ values cannot be accurately determined because the binding is not saturated even at the highest protein concentration.

To measure the binding affinity between ASH2L and RBBP5, ASH2L (or the ASH2L-DPY30 complex) was diluted to a series of concentrations from 20 μM to 0.01 μM in buffer containing 25 mM Tris, pH 8.0, and 150 mM NaCl. Fifteen microliters of various concentrations of proteins were mixed with equal volumes of 200 nM RBBP5_330-363_-FAM and incubated on ice in the dark for 30 min. The following experimental operations were consistent with those mentioned above.

#### Electrophoretic mobility shift assay (EMSA)

ASH2L or the ASH2L-DPY30 complex was diluted to a series of concentrations ranging from 11.52 μM to 0.18 μM using buffer containing 25 mM HEPES pH 7.4, 150 mM NaCl, 0.5 mg/mL BSA, and 5% glycerol.7 μL of various concentrations of protein was mixed with 7 μL of 80 nM 25 bp 5′-FAM-labeled dsDNA (FAM-TCTCTAGAGTCGACCTGCAGGCATG). After incubation on ice for 30 min, each reaction mixture was separated by electrophoresis on a 6% native polyacrylamide gel. The gels were then visualized on a Bio-Rad ChemiDoc MP Imaging System (Bio-Rad, USA).

#### Structural prediction by ColabFold

Structural prediction was carried out by ColabFold, which was powered by AlphaFold2 and featured sequence alignment using MMseqs2 (Mirdita et al., 2022). The parameters were the default settings, including unrelaxed, no template information used, MMseqs2 (UniRef + Environment), pair mode (unpaired + paired), and model_type (complex prediction using Alpha-Fold-multimer-v2 and single-chain prediction using AlphaFold2-ptm). The results were similar to the models predicted by AlphaFold2 v2.0.0 (Jumper et al., 2021) installed in the local workstation using the nondocker installation. The output five models were aligned and inspected in PyMOL (Schrödinger, USA).

#### Molecular dynamics simulations

For MD simulation, apo ASH2L_230-534_ and the ASH2L_230-534_-DPY30_DD_ complex were used because the turbulent motion between ASH2L_1-177_ and ASH2L_230-534_ because of the highly flexible ASH2L_IDR_ (residues 178-229) leads to overall dynamics in ASH2L_FL_. All systems were set up using GROMACS (Kutzner et al., 2015) and the CHARMM36 force field (Huang and MacKerell, 2013). The proteins were centered in a cubic box with a buffering distance of 1.0 nm and solvated with TIP3P water molecules. Charges were neutralized by adding Na^+^ or Cl^−^ accordingly, and the final NaCl concentration was kept at 0.15 M, consistent with the experiments. After energy minimization with the steepest descending algorithm, we gently heated the system to 300 K under NVT conditions with the position of the protein constrained with a harmonic potential of 1000 kJ/mol. Then, the NPT ensemble was carried out with the position of the protein constrained. A V-rescale thermostat (Bussi et al., 2007) was used to control the temperature with a time constant of 0.1 ps, and a Berendsen barostat was used to control the pressure at 1 bar with a coupling constant of 2 ps. After equilibration, we switched the temperature coupling to Parrinello-Rahman and pressure coupling to Nose–Hoover with coupling constants of 5 and 1 ps, respectively. Molecular dynamics simulation systems were investigated for different combinations of proteins with a simulation time of 100 ns. The simulation lasted for approximately 24 h on an i9-9900X with an RTX 3080. The finished simulation trajectory was corrected for periodic boundary conditions using the *trjconv* module in GROMACS.

The secondary structure of ASH2L during molecular dynamics simulation was calculated based on the simulation trajectory in VMD 1.9.1 (Humphrey et al., 1996). Based on the official DSSP standard (Joosten et al., 2010; Kabsch and Sander, 1983), we simplified the secondary structure into three main categories, namely, 1. α-helix, which contains 3-helix (G), 5-helix (I), and the standard α-helix (H); 2. β-strand, which contains residue in isolated β-bridge (B) and extended strand, participates in β-ladder(E); 3. unstructured, which contains hydrogen-bonded turn (T), bend (S), and blank in DSSP secondary structure (C). The output secondary structure data from VMD were replotted using a Python script with the NumPy, Seaborn, and Matplotlib packages in Anaconda3.

#### Biological small-angle X-Ray scattering

Small-angle X-ray scattering (SAXS) experiments were performed at beamline BL19U2 of the National Facility for Protein Science Shanghai (NFPS) at Shanghai Synchrotron Radiation Facility (SSRF). The wavelength λ of X-ray radiation was set as 0.918 Å. Scattered X-ray intensities were collected using a Pilatus 1M detector (DECTRIS Ltd, Switzerland). The sample-to-detector distance was set such that the detecting range of momentum transfer [*q* = 4π sinθ/λ, where 2θ is the scattering angle] of SAXS experiments was 0.008-0.47 Å^−1^. A flow cell made of a cylindrical quartz capillary with a diameter of 1.5 mm and a wall of 10 μm was used. M1WRA and M1WRAD complexes were diluted to three different concentrations (0.5, 1, and 2 mg/mL) in buffer containing 25 mM HEPES pH 7.4, 150 mM NaCl, and 1 mM TCEP. SAXS data were collected as 20 × 1 s exposures, and scattering profiles for the 20 passes were compared at 10°C using a 60 μL sample. The 2D scattering images were converted to 1D SAXS curves through azimuthally averaging after solid angle correction and then normalizing with the intensity of the transmitted X-ray beam using the software package BioXTAS RAW (Hopkins et al., 2017). Background scattering was subtracted using PRIMUS in the ATSAS software package (Franke et al., 2017). Linear Guinier plots in the Guinier region (*q*∗*R*_g_< 1.3) were confirmed in all experimental groups. Pair distance distribution functions of the particles *P*(r) and the maximum sizes *d*_max_ were computed using GNOM (Svergun, 1992).

### Quantification and statistical analysis

All data are shown as the mean ± SD from three independent experiments. Statistical analysis was performed by two-tailed Student’s t test using GraphPad Prism 8.0 software. Values of p < 0.05 were considered statistically significant. Levels of significance are expressed as follows: ns, not significant; ∗, 0.01 < p < 0.05; ∗∗, 0.001 < p < 0.01; ∗∗∗, 0.0001 < p < 0.001; ∗∗∗∗, p < 0.0001.

## Data Availability

• Raw SDS-PAGE gels and western blot images have been deposited at Mendeley Data and can be accessed through the https://doi.org/10.17632/s9g2mr6ytx.1 (https://data.mendeley.com/datasets/s9g2mr6ytx/1). • The SAXS data have been deposited at SASDBD (https://www.sasdbd.org/) and are publicly available as the date of publication. The accession codes SASDPT3 (M1WRA) and SASDPU3 (M1WRAD) are listed in the key resources table. • This paper does not report original code. • Any additional information required to reanalyze the data reported in this paper is available from the lead contact on request.

## Supplemental information

Document S1. Figures S1–S8 and Tables S1–S3

Data S1. The complete list of the crosslinked peptides identified in the M1WRA and M1WRAD samples, related to Figure 2 and Table S1

Data S2. The complete list of the crosslinked peptides identified in the M1WRA + NCP and M1WRAD + NCP samples, related to Figure 6 and Table S3
