# Supplementary material for: DPY30 acts as an ASH2L-specific stabilizer to stimulate the enzyme activity of MLL family methyltransferases on different substrates
Source: iScience. 2022 Aug 16;25(9):104948. doi: 10.1016/j.isci.2022.104948 (PMC9440282; doi:10.1016/j.isci.2022.104948)
Supplement: Document S1. Figures S1–S8 and Tables S1–S3 [file mmc1.pdf]

iScience, Volume *25*

Supplemental information

DPY30 acts as an ASH2L-specific stabilizer to stimulate the enzyme activity of MLL family methyltransferases on different substrates

Lijie Zhao, Naizhe Huang, Jun Mencius, Yanjing Li, Ying Xu, Yongxin Zheng, Wei He, Na Li, Jun Zheng, Min Zhuang, Shu Quan, and Yong Chen

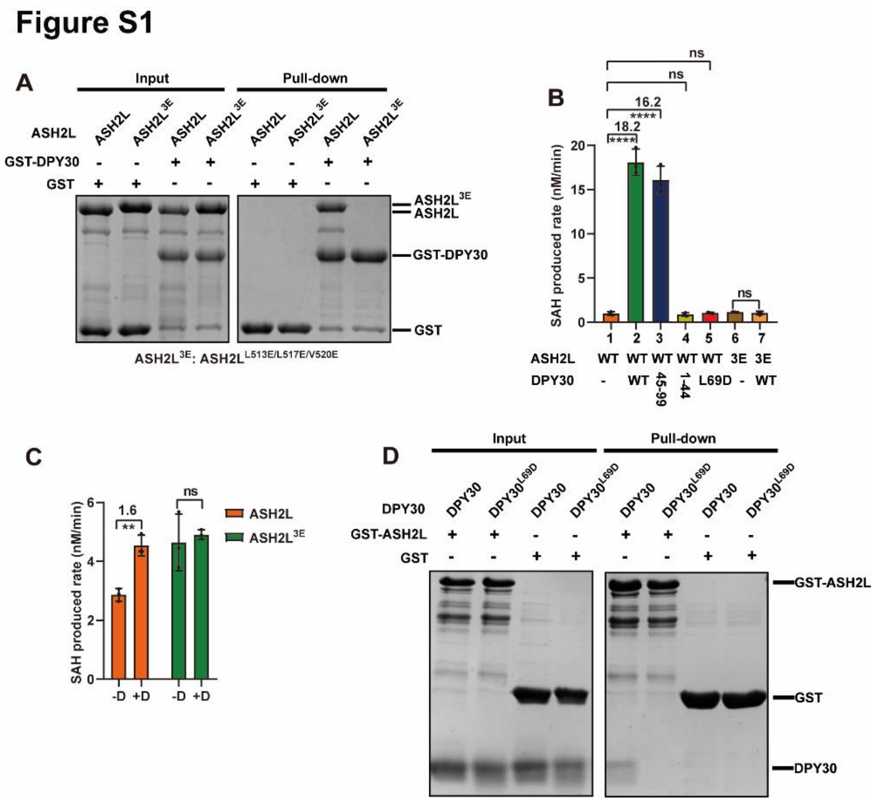

Figure S1. The activity-stimulating effect of DPY30 relies on its interaction with ASH2L. Related to Figure 2.

A. GST pull-down assays revealed that ASH2L$^{L513E/L517E/V520E}$ (ASH2L^{3E}) could disrupt the ASH2L-DPY30 interaction. GST-DPY30 was incubated with ASH2L or its mutant as indicated on the top. Bound proteins were eluted and separated by SDS–PAGE.

B. Comparison of NCP methylation rates of MLL1 complexes reconstituted with different ASH2L/DPY30 truncations or mutations. The ratio of the methylation rate of M1WRAD to the methylation rate of M1WRA is indicated on the top. Data are shown as the mean ± s.d. from triplicate measurements.

C. Comparison of $H3_{1\text{-}9}$ methylation rates of MLL1 complexes reconstituted with wild-type ASH2L (WT) and mutated ASH2L (ASH2L^{3E}). The ASH2L-DPY30 interaction is also important for the methylation of H3 peptides.

D. GST pull-down assays revealed that DPY30^{L69D} could disrupt the ASH2L-DPY30 interaction.

Figure S2

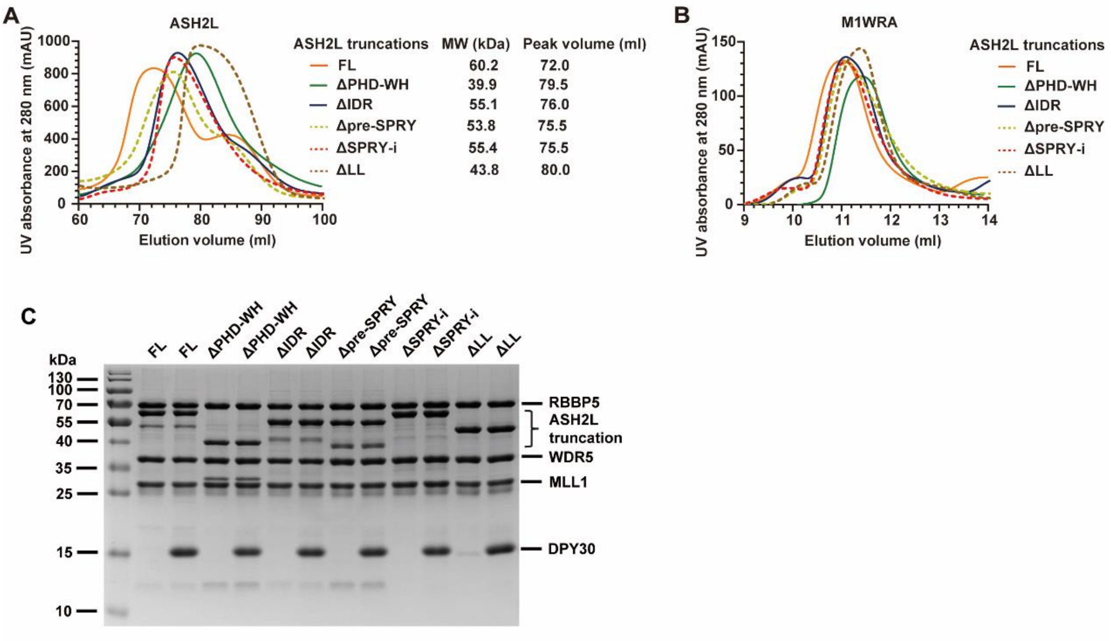

Figure S2. Structural integrity analyses of different ASH2L mutants. Related to Figure 2.

A. Comparison of gel filtration profiles of different ASH2L domain-deletion mutants. The molecular weight and peak positions of different ASH2L mutants are shown on the right.

B. Comparison of gel filtration profiles of the M1WRA complex assembled with different ASH2L mutants.

C. SDS–PAGE analysis of different MLL1 complexes assembled with different ASH2L domain-deletion mutants. For each ASH2L deletion mutant, the left lane is the preassembled M1WRA complex, and the right lane is M1WRA+DPY30 (DPY30 was added to the preassembled M1WRA complex).

Figure S3

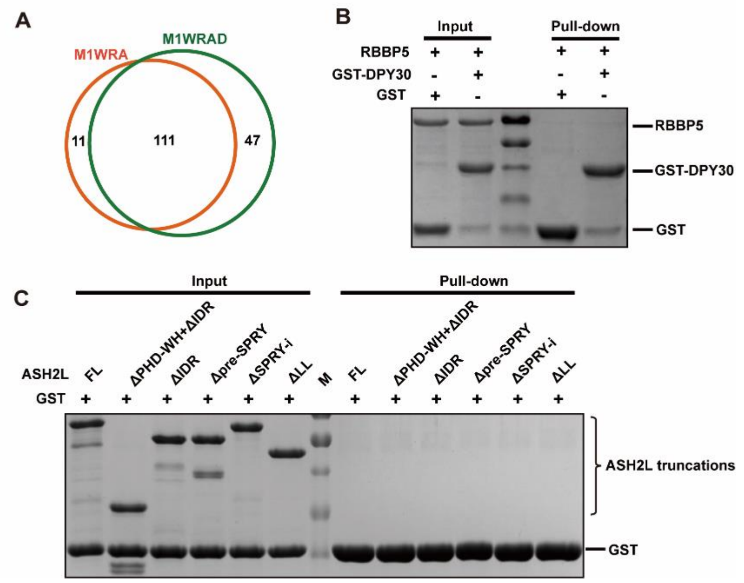

Figure S3. Validation of the crosslinking mass spectrometry results. Related to Figure 2.

A. Venn diagram of the peptide crosslinks identified in M1WRA and M1WRAD. The cutoff of spectrum counts of more than three and an E score value smaller than 0.02 was used to sort the data obtained from DSS-crosslinking mass spectrometry.

B. GST pull-down assays revealed that DPY30 could not interact with RBBP5. GST-DPY30 was incubated with RBBP5, and bound proteins were eluted and separated by SDS–PAGE.

C. GST pull-down assays showed that all ASH2L truncations had no nonspecific binding with the GST beads. The GST tag was incubated with different ASH2L truncations as indicated on the top. Bound proteins were eluted and separated by SDS–PAGE.

Figure S4

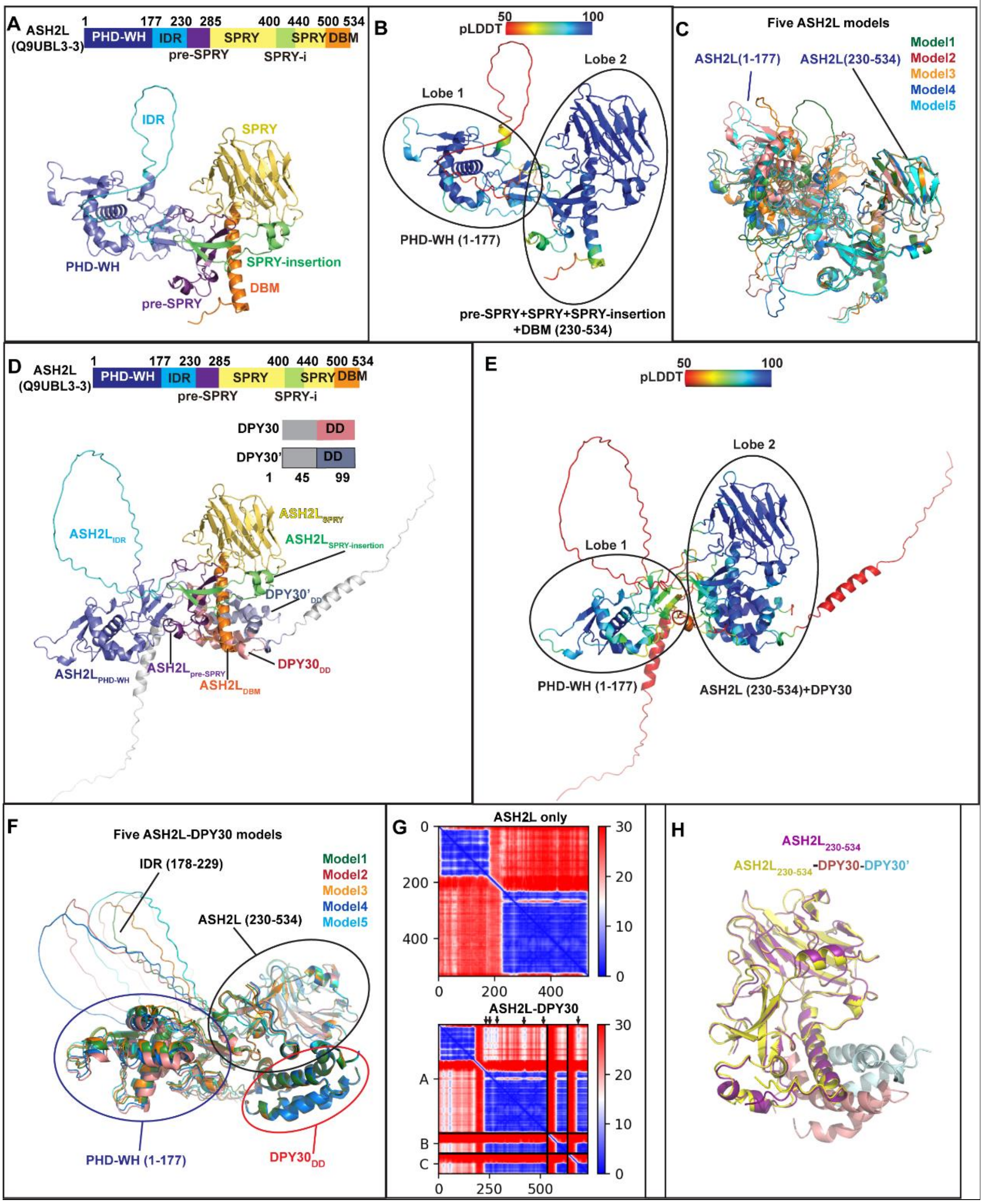

Figure S4. Structure of ASH2L-DPY30 predicted by ColabFold. Related to Figure 3.

A. The highest-ranked model of ASH2L predicted by ColabFold. Different ASH2L domains or motifs are in the following color codes: $ASH2L_{PHD-WH}$, dark blue; $ASH2L_{IDR}$, cyan; $ASH2L_{pre-SPRY}$, magenta; $ASH2L_{SPRY}$, yellow; $ASH2L_{SPRY-insertion}$, green; $ASH2L_{DBM}$, orange.

B. pLDDT values of ASH2L predicted by ColabFold shown in cartoon mode. Model confidence: very high (pLDDT>90); confident (90>pLDDT>70); low (70>pLDDT>50); very low (pLDD<50).

Most regions of ASH2L are structured with high confidence, except for a large IDR loop (178-229), the very N-terminal loop (1-12), and the very C-terminal loop (526-534).

C. Comparison of the five highest-ranked models of ASH2L-DPY30. These five models are aligned by $ASH2L_{230-534}$, which can be superimposed with rmsd less than 0.5 Å. The PHD-WH domains of ASH2L (1-177) showed different orientations relative to the aligned $ASH2L_{230-534}$ due to the flexible IDR loop (178-229). Five models are shown in cartoon mode of different colors.

D. The highest-ranked model of the ASH2L-DPY30 complex predicted by ColabFold. Different ASH2L domains or motifs are in the same color codes as in panel A. Two DPY30 are colored in light blue and red, respectively.

E. pLDDT values of ASH2L-DPY30 predicted by ColabFold shown in cartoon mode. Most regions of ASH2L and DPY30 are structured with high confidence. The following regions are unstructured: ASH2L N-terminal loop (1-12), ASH2L IDR loop (178-229), ASH2L C-terminal loop (526-534), and DPY30 N-terminal IDR loop (1-45).

F. Comparison of the five highest-ranked models of ASH2L-DPY30. These five models are aligned by $ASH2L_{230-534}$, which can be superimposed with rmsd less than 0.5 Å. The PHD-WH domains of ASH2L (1-177) showed similar orientation relative to the aligned $ASH2L_{230-534}$, partly due to the newly formed $ASH2L_{PHD-WH}$-DPY30 interface. Five models are shown in cartoon mode of different colors.

G. PAE (predicted aligned error) plots for apo ASH2L and ASH2L-DPY30. The color at position (x, y) indicates the expected position error at residue x when the predicted and true structures are aligned on residue y. Compared with ASH2L, ASH2L-DPY30 has some low PAE regions (blue colored regions, labeled with arrows), indicating some interdomain packings.

H. Superimposition of the apo $ASH2L_{230-534}$ and $ASH2L_{230-534}$-DPY30 complexes. DPY30 does not change the conformation of ASH2L because these two structures can be superimposed with a rmsd value of 0.32 Å.

Figure S5

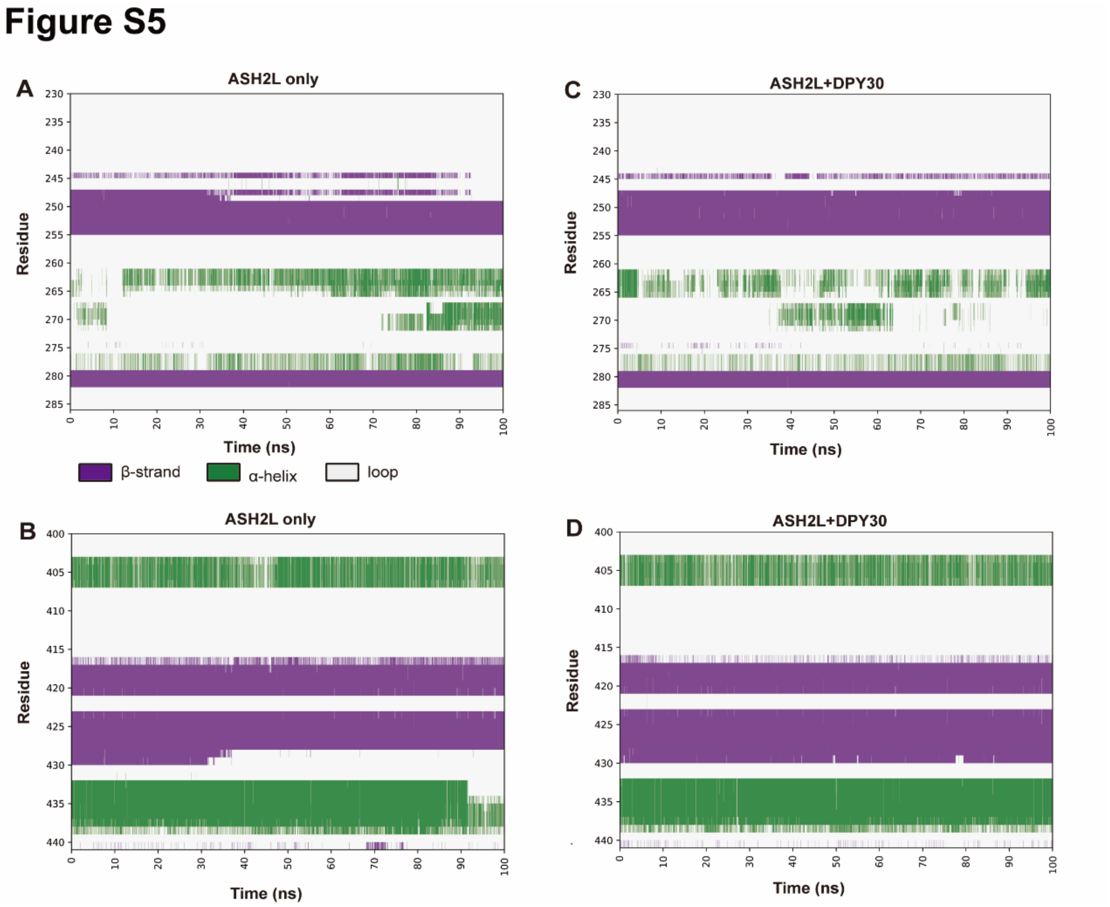

Figure S5. Secondary structures of ASH2L during molecular dynamics simulations. Related to Figure 3.

The α-helix, β-strand, and unstructured regions of ASH2L during molecular dynamic simulation are colored purple, green, and gray, respectively, in the heatmap. For all heatmaps, the Y-axis is the residue span of ASH2L. The X-axis is the span of the simulation time.

A. The secondary structure stability of the $ASH2L_{pre\text{-}SPRY}$ motif (residues 230-285) in apo ASH2L.

B. The secondary structure stability of the $ASH2L_{SPRY\text{-}insertion}$ motif (residues 400-440) in apo ASH2L.

C. The secondary structure stability of the $ASH2L_{pre\text{-}SPRY}$ motif (residues 230-285) in the ASH2L-DPY30 complex.

D. The secondary structure stability of the $ASH2L_{SPRY\text{-}insertion}$ motif (residues 400-440) in the ASH2L-DPY30 complex.

Figure S6

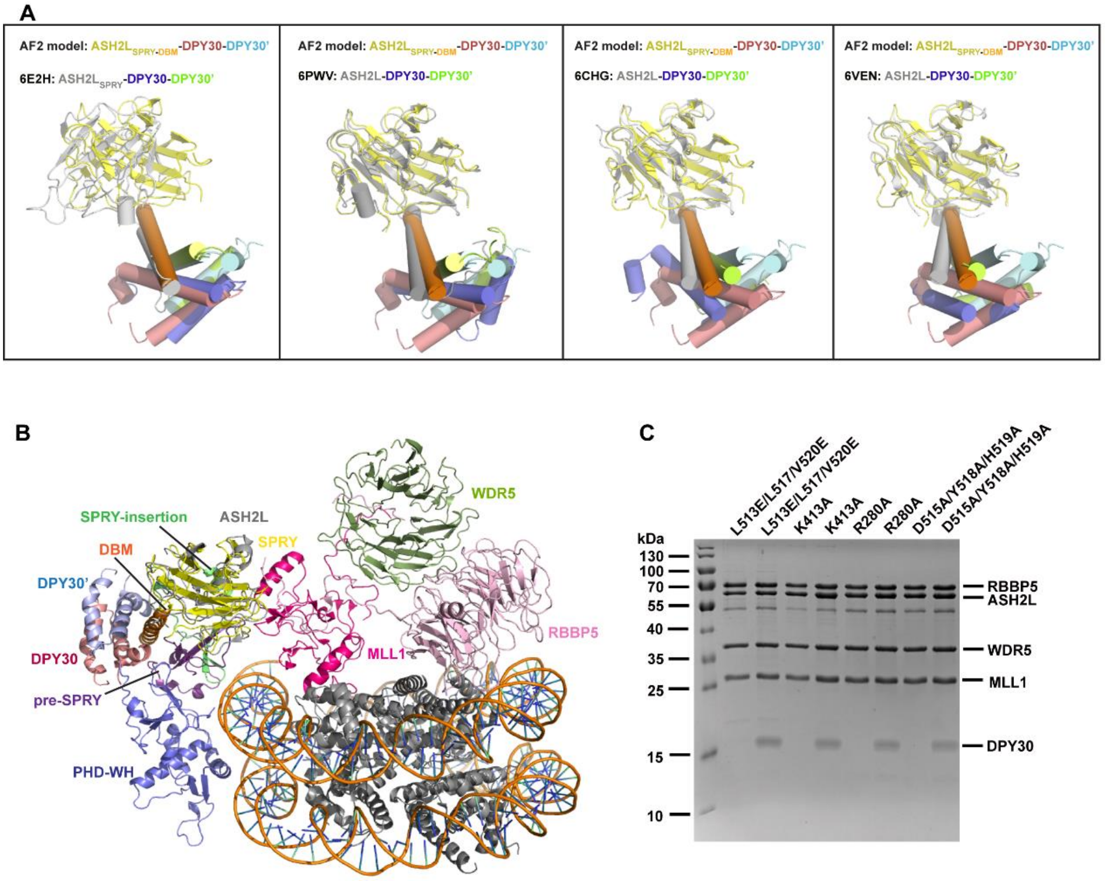

Figure S6. Structural comparison of our ASH2L-DPY30 model with the previously determined ASH2L-DPY30 or Bre2-Sdc1 structures. Related to Figure 3.

A. Superimposition of the AlphaFold2-predicted ASH2L-DPY30 structure with ASH2L-DPY30 in 6E2H, 6PWV, 6CHG, and 6VEN. For clarity, the pre-SPRY and SPRY-insertion motifs of ASH2L are not shown, and the unstructured N-terminus of DPY30 is not shown. In the AlphaFold2 model, $ASH2L_{SPRY}$ is colored yellow, with the DBM helix in orange, and two DPY30 are colored red and cyan. In the 6E2H, 6PWV, 6CHG, and 6VEN models, ASH2L (or Bre2) are colored in gray, and two DPY30 (or Sdc1) are colored in blue and green.

B. Superimposition of the predicted ASH2L-DPY30 structure and the cryo-EM structure of M1WRA-NCP (PDB: 6KIX). In the AlphaFold2 model, different ASH2L motifs are differentially colored: PHD-WH, blue; pre-SPRY, purple; SPRY, yellow; SPRY-insertion, dark green; DBM, orange. Two DPY30 molecules are colored in light blue and dark red. In the 6KIX structure, ASH2L is colored grey, WDR5 is colored green, RBBP5 is colored pink, and MLL1 is colored magenta. The predicted ASH2L-DPY30 structure could be fit into the determined cryo-EM

structure without any steric clash. For clarity, the highly flexible $ASH2L_{IDR}$ (178-229) and $DPY30_{IDR}$ (1-45) are not shown because they don’t have defined configurations.

C. SDS-PAGE analysis of different MLL1 complexes assembled with different ASH2L mutants. For each ASH2L mutant, the left lane is the preassembled M1WRA complex, and the right lane is M1WRA+DPY30 (DPY30 was added to the preassembled M1WRA complex).

Figure S7

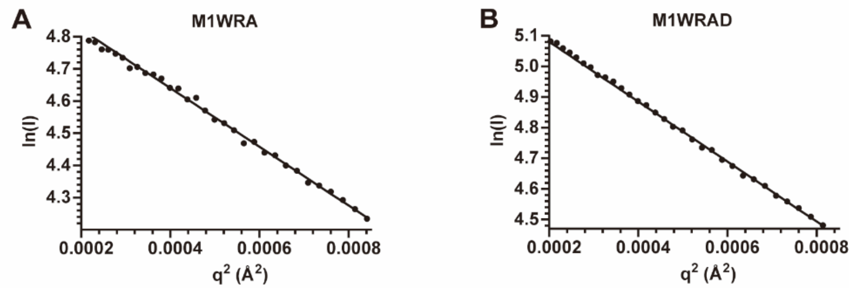

Figure S7. Small-angle X-ray scattering analyses of the MLL1 complex. Related to Figure 5.

The Guinier plots of the scattering data show a linear fit at low *q*. The SAXS data shown were from the 2 mg/mL M1WRA complex (A) and the M1WRAD complex (B).

Figure S8

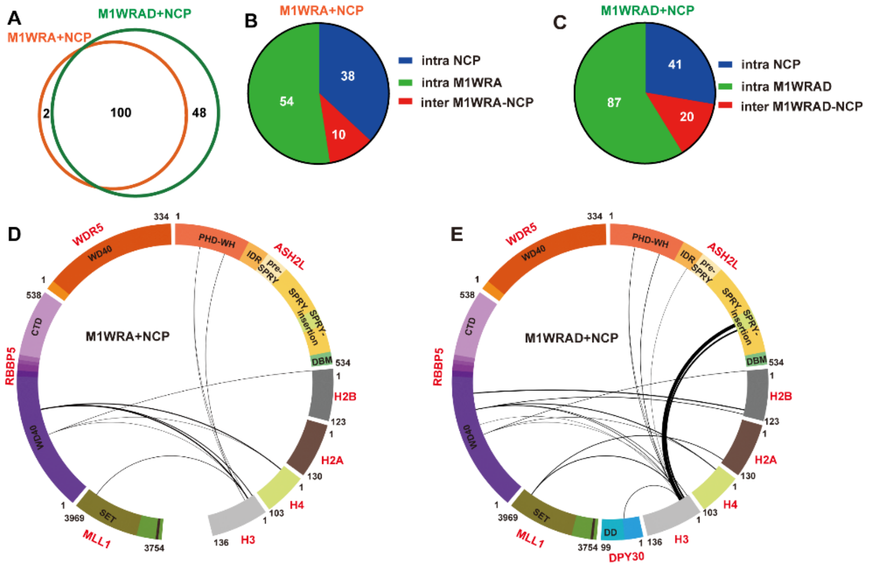

Figure S8. Crosslinking mass spectrometry analyses of M1WRAD+NCP and M1WRA+NCP. Related to Figure 6.

A. Venn diagram of the peptide crosslinks identified in M1WRA+NCP and M1WRAD+NCP. The cutoff of spectrum counts of more than three and an E score value smaller than 0.02 was used to sort the data obtained from DSS-crosslinking mass spectrometry.

B. Pie chart showing the numbers of 102 M1WRA+NCP crosslinks in three categories: crosslinks inside the NCP (intra NCP), crosslinks inside the MLL1 complex (intra M1WRA), and intersubunit crosslinks between the MLL1 complex and NCP (inter M1WRA-NCP).

C. Pie chart showing the numbers of M1WRAD+NCP crosslinks in the three categories.

D. Circular plot of crosslinks between the MLL1 complex and NCP detected in the M1WRA+NCP sample. The thickness of the line is correlated to the spectrum number of the crosslinked peptide detected.

E. Circular plot of crosslinks between the MLL1 complex and NCP detected in the M1WRAD+NCP sample.

Table S1. Specific crosslinks found only in M1WRAD but not in M1WRA. Related to Figure 2.

Protein1(site)-Protein2(site)	#Spec-Total	Best Score
ASH2L(101)-ASH2L(419)	3	6.44E-03
ASH2L(109)-DPY30(35)	6	4.09E-04
ASH2L(131)-ASH2L(419)	32	1.19E-03
ASH2L(131)-ASH2L(434)	3	1.48E-02
ASH2L(131)-DPY30(40)	3	1.02E-02
ASH2L(135)-DPY30(40)	6	8.98E-04
ASH2L(135)-DPY30(45)	5	1.86E-02
ASH2L(135)-DPY30(90)	4	2.03E-03
ASH2L(135)-DPY30(92)	5	3.92E-03
ASH2L(151)-ASH2L(419)	7	1.95E-03
ASH2L(151)-DPY30(45)	3	4.70E-03
ASH2L(207)-DPY30(35)	15	1.62E-04
ASH2L(207)-DPY30(40)	5	6.03E-03
ASH2L(207)-DPY30(45)	10	1.70E-05
ASH2L(207)-DPY30(90)	9	7.72E-07
ASH2L(207)-DPY30(92)	6	2.09E-03
ASH2L(218)-ASH2L(419)	8	4.46E-03
ASH2L(244)-ASH2L(311)	14	9.47E-03
ASH2L(244)-MLL1(3924)	14	9.68E-05
ASH2L(406)-DPY30(45)	5	1.63E-04
ASH2L(419)-DPY30(40)	10	1.68E-02
ASH2L(419)-RBBP5(505)	5	1.29E-02
ASH2L(434)-DPY30(35)	13	5.57E-05
ASH2L(440)-DPY30(35)	3	2.46E-03
ASH2L(440)-DPY30(40)	12	8.08E-06
ASH2L(461)-DPY30(35)	3	1.42E-03

ASH2L(67)-DPY30(40)	4	1.48E-04
ASH2L(67)-DPY30(90)	4	1.07E-03
ASH2L(67)-DPY30(92)	4	1.45E-06
ASH2L(67)-MLL1(3772)	3	5.48E-03
ASH2L(67)-MLL1(3804)	3	5.86E-03
ASH2L(99)-ASH2L(419)	8	1.18E-03
DPY30(35)-DPY30(45)	21	1.01E-05
DPY30(35)-DPY30(90)	8	7.51E-07
DPY30(35)-DPY30(92)	15	5.01E-04
DPY30(35)-RBBP5(244)	3	5.69E-03
DPY30(40)-DPY30(45)	17	1.16E-05
DPY30(40)-DPY30(90)	11	1.10E-04
DPY30(40)-DPY30(92)	9	1.06E-04
DPY30(40)-RBBP5(174)	4	1.70E-04
DPY30(40)-RBBP5(244)	3	7.87E-03
DPY30(45)-DPY30(45)	3	6.34E-04
DPY30(45)-DPY30(90)	7	3.24E-03
DPY30(45)-DPY30(92)	28	3.20E-07
DPY30(45)-RBBP5(244)	5	2.19E-04
DPY30(90)-DPY30(92)	11	1.04E-05
RBBP5(174)-RBBP5(281)	3	7.89E-03

Table S2. Summary of SAXS analyses of the M1WRA and M1WRAD complexes. Related to Figure 5.

(a) Sample details

	M1WRA	M1WRAD
Organism	*Homo sapiens*	*Homo sapiens*
Source (Catalog No. or reference)	*E.coli expressed*	*E.coli expressed*
UniProt sequence ID (residues in construct)	Q03164 (3754-3969)<br>P61964 (1-334)<br>Q15291 (1-538)<br>Q9UBL3-3 (1-534)	Q03164 (3754-3969)<br>P61964 (1-334)<br>Q15291 (1-538)<br>Q9UBL3-3 (1-534)<br>Q9C005 (1-99)
Extinction coefficient ε (wavelength and units) (A280, M^{-1} cm^{-1})	237970	245650
Molecular mass *M* from chemical composition (KDa)	180.9	203.3
Concentration (mg/ml)	0.5-2	0.5-2
Solvent composition and source	150 mM NaCl, 25 mM Tris pH 8.0, 1 mM TCEP	

(b) SAS data collection parameters

SAS data collection parameters Source, instrument and description or reference	BL19U2 at SSRF with Pilatus 1 M (DECTRIS Ltd)
Wavelength (Å)	0.9184
Beam geometry (size, sample-to-detector distance)	340 μm x 60 μm (H x V), 2.415 m
q-measurement range ($Å^{-1}$)	0.008-0.47
Absolute scaling method	Comparison with scattering with 1 mm pure H2O
Basis for normalization to constant counts	Take silver behenate as standard to set the mask, then normalize the 2D images
Method for monitoring radiation damage, X-ray dose where relevant	SAXS data were collected as continuous serial exposures and scattering profiles for the passes were compared to monitor the radiation damage
Exposure time, number of exposures	1 s per frame, total 20 frames
Sample temperature (°C)	10

(c) Software employed for SAS data reduction, analysis and interpretation

SAS data reduction	*ln*(*I*) versus *q2* using OriginPro 9.0, solvent substraction using *PRIMUS* (*ATSAS* 3.0.3)
Extinction coefficient estimate	http://protcalc.sourceforge.net/
Basic analyses: Guinier, *P*(r), *V*p	*PRIMUS* (*ATSAS* 3.0.3)

(d) Structural parameters

	M1WRA	M1WRAD
Guinier Analysis		
$I(0)$ (cm^{-1})	149.22±1.64	195.67±1.42
R_g (Å)	52.33±0.48	54.18±0.31
q-range ($Å^{-1}$)	0.0147-0.0290	0.0143-0.0285
Quality-of-fit parameter (fidelity)	0. 86	0.88
P(r) analysis		
$I(0)$ (cm^{-1})	149.00±0.10	192.40±0.80
R_g (Å)	54.42±0.29	54.73±0.17
d_{max} (Å)	185.00	175.00
q-range ($Å^{-1}$)	0.0128-0.2921	0.0128-0.2921
Quality-of-fit parameter (Total estimate from *GNOM*)	0.85	0.84
Porod volume estimate ($Å^3$)	385767	410857

(e) Data and model deposition IDs

	M1WRA	M1WRAD
SASDBD (https://www.sasbdb.org)	SASDPT3	SASDPU3

Table S3. Specific crosslinks found only in the M1WRAD-NCP sample but not in the M1WRA-NCP sample. Related to Figure 6.

Protein1(site)-Protein2(site)	#Spec-Total	Best Score
ASH2L(109)-ASH2L(122)	3	4.51E-03
ASH2L(109)-ASH2L(419)	5	7.42E-03
ASH2L(109)-DPY30(45)	3	2.45E-04
ASH2L(135)-DPY30(40)	3	1.67E-02
ASH2L(151)-ASH2L(419)	7	7.17E-04
ASH2L(207)-ASH2L(421)	4	1.75E-05
ASH2L(207)-DPY30(35)	10	9.31E-04
ASH2L(207)-DPY30(45)	10	2.14E-04
ASH2L(207)-DPY30(90)	3	2.76E-06
ASH2L(218)-ASH2L(419)	8	1.31E-04
ASH2L(218)-H3(18)	3	1.57E-02
ASH2L(272)-ASH2L(419)	5	6.39E-03
ASH2L(361)-ASH2L(440)	3	4.70E-04
ASH2L(419)-RBBP5(174)	3	1.46E-03
ASH2L(419)-H3(23)	42	4.96E-04
ASH2L(419)-H3(27)	15	9.87E-06
ASH2L(421)-H3(14)	3	6.84E-05
ASH2L(434)-DPY30(35)	7	1.07E-02
ASH2L(434)-H3(14)	19	5.24E-05
ASH2L(440)-DPY30(40)	13	6.42E-05
ASH2L(461)-DPY30(35)	4	7.54E-03
ASH2L(67)-DPY30(40)	3	1.36E-02
ASH2L(67)-DPY30(90)	6	9.83E-03
ASH2L(67)-DPY30(92)	4	1.46E-03
ASH2L(67)-MLL1(3924)	3	1.09E-02
DPY30(35)-DPY30(45)	22	4.66E-08

DPY30(35)-DPY30(90)	8	8.11E-08
DPY30(35)-DPY30(92)	19	2.42E-07
DPY30(40)-DPY30(45)	21	1.26E-06
DPY30(40)-DPY30(90)	9	2.72E-04
DPY30(40)-DPY30(92)	13	2.02E-05
DPY30(40)-H3(27)	6	9.40E-06
DPY30(45)-DPY30(90)	6	1.07E-03
DPY30(45)-DPY30(92)	30	7.31E-09
DPY30(45)-RBBP5(244)	5	7.67E-04
DPY30(90)-DPY30(92)	12	3.28E-08
MLL1(3945)-H2A(119)	8	1.37E-06
RBBP5(172)-RBBP5(482)	3	4.28E-03
RBBP5(174)-RBBP5(488)	6	1.20E-04
RBBP5(202)-H3(27)	3	1.76E-02
RBBP5(244)-RBBP5(288)	4	9.68E-07
RBBP5(244)-H2B(117)	7	8.46E-03
RBBP5(288)-H2B(105)	12	1.04E-03
H2A(95)-H4(59)	5	2.27E-03
H2B(1)-H2B(54)	8	6.75E-04
H2B(31)-H2B(54)	4	7.68E-07
H3(56)-H4(91)	4	5.33E-07
H4(12)-H4(59)	3	1.70E-02
